# Sequence-agnostic motion-correction leveraging efficiently calibrated Pilot Tone signals

**DOI:** 10.1002/mrm.30161

**Published:** 2024-06-11

**Authors:** Yannick Brackenier, Lucilio Cordero-Grande, Sarah McElroy, Raphael Tomi-Tricot, Hugo Barbaroux, Philippa Bridgen, Shaihan J. Malik, Joseph V. Hajnal

**Affiliations:** 1 Biomedical Engineering Department, School of Biomedical Engineering and Imaging Sciences, King’s College London, London, UK; 2 Center for the Developing Brain, School of Biomedical Engineering and Imaging Sciences, King’s College London, London, UK; 3 Biomedical Image Technologies, ETSI Telecomunicación, Universidad Politécnica de Madrid and CIBER-BNN, ISCIII, Madrid, Spain; 4 MR Research Collaborations, Siemens Healthcare Limited, Frimley, UK

**Keywords:** motion correction, parallel imaging, Pilot Tone (PT), reconstruction, ultrahigh field

## Abstract

**Purpose:**

This study leverages externally generated Pilot Tone (PT) signals to perform motion-corrected brain MRI for sequences with arbitrary k-space sampling and image contrast.

**Theory and Methods:**

PT signals are promising external motion sensors due to their cost-effectiveness, easy workflow, and consistent performance across contrasts and sampling patterns. However, they lack robust calibration pipelines. This work calibrates PT signal to rigid motion parameters acquired during short blocks (~4 s) of motion calibration (MC) acquisitions, which are short enough to unobstructively fit between acquisitions. MC acquisitions leverage self-navigated trajectories that enable state-of-the-art motion estimation methods for efficient calibration. To capture the range of patient motion occurring throughout the examination, distributed motion calibration (DMC) uses data acquired from MC scans distributed across the entire examination. After calibration, PT is used to retrospectively motion-correct sequences with arbitrary k-space sampling and image contrast. Additionally, a data-driven calibration refinement is proposed to tailor calibration models to individual acquisitions. In vivo experiments involving 12 healthy volunteers tested the DMC protocol’s ability to robustly correct subject motion.

**Results:**

The proposed calibration pipeline produces pose parameters consistent with reference values, even when distributing only six of these approximately 4-s MC blocks, resulting in a total acquisition time of 22 s. In vivo motion experiments reveal significant (*p* < 0.05) improved motion correction with increased signal to residual ratio for both MPRAGE and SPACE sequences with standard k-space acquisition, especially when motion is large. Additionally, results highlight the benefits of using a distributed calibration approach.

**Conclusions:**

This study presents a framework for performing motion-corrected brain MRI in sequences with arbitrary k-space encoding and contrast, using externally generated PT signals. The DMC protocol is introduced, promoting observation of patient motion occurring throughout the examination and providing a calibration pipeline suitable for clinical deployment. The method’s application is demonstrated in standard volumetric MPRAGE and SPACE sequences.

## 1 Introduction

MRI is sensitive to motion that causes artifacts in reconstructed images, especially with prolonged scanning times for high-resolution imaging.^1,2^ Both prospective and retrospective motion-correcting schemes have been proposed,^3,4^ the latter aiming to correct data acquisition in real time, and the former aiming to correct image reconstruction based on estimates of the patient’s motion history. Motion correction for brain imaging is usually performed with a rigid body approximation.

External measurement systems can provide information on motion that is independent of the NMR signal/-contrast or k-space sampling,^5–9^ allowing use with almost any sequence. Pilot Tone (PT) signals are one example, whereby RF signals close to the NMR resonance frequency are injected into the scanner room and detected by every receiver coil without interfering with the NMR signal.^10,11^ Motion of the patient’s body modulates these signals, hence encoding motion information. Although originally introduced to track cardiac and respiratory motion, PT signals can also encode head motion, in which signals are modulated differentially between receiver elements.^12,13^ Because the PT signals are directly detected by the MR scanner and incorporated into the scan data, the method provides a cheap and streamlined workflow with measurements automatically synchronized with the MR signals.

PT signals do not explicitly encode motion parameters. Instead, it is usually necessary to define a mapping between the measured signals and the motion parameters (i.e., three translation and three rotations for rigid body motion). This can be achieved by acquiring data at the start of the examination.^12,14–16^ A common solution is to execute a training phase (called *precalibration*), during which fast 3D imaging data and PT signals are simultaneously acquired in different static poses. Registering the reconstructed images provides pose parameters that can then be used to calibrate the PT signals. To capture sufficient pose information, subjects are asked to repeat this experiment in different poses. Although successful implementations exist,^14,15^ this approach has limitations. First, the instructed motion does not necessarily capture the full range of poses observed across an entire examination. Second, relying on instructed motion would not integrate easily into clinical workflows. Finally, registering the reconstructed images for pose estimation may not be the most efficient calibration strategy; only one set of six pose parameters is obtained from each (fast) 3D image acquisition. Ideally, within-acquisition motion can be used to obtain more pose parameters and hence improve calibration efficiency.

Alternatively, motion information can be obtained directly from acquired k-space samples using the patient’s NMR signal. Navigators are dedicated sequence-encoding blocks added to the sequence and encode motion-induced changes in k-space (“navigator k-space”). For brain MRI, a wide variety of navigator trajectories exist, accompanied with strategies to retrieve the rigid motion parameters.^17–25^ Nevertheless, in most cases, the stability and/or accuracy needed for clinical deployment have not yet been established. Self-navigation directly uses k-space samples acquired for image reconstruction (“imaging k-space”) to estimate rigid motion. Self-navigated trajectories can be obtained by re-ordering the standard phase-encoding k-space trajectory and can avoid increasing the total acquisition time (TA), making them time-efficient. Combining self-navigated trajectories (e.g., DISORDER, PROPELLER, Linear^+^^26–28^) with data-driven motion and/or image estimation algorithms (e.g., SAMER, alignedSENSE^27,29,30^) has shown excellent performance and is getting validated for clinical use.^31,32^ However, self-navigation requires sequence modifications and can restrict sequence design (e.g., contrast layout), making it challenging to obtain images corresponding to the original/unmodified protocols. On the other hand, “standard” acquisitions (e.g., using linear phase-encoding sampling) do not always achieve stable motion correction and can lead to uncorrectable data.

In this work, we aim to achieve versatile motion correction leveraging the best of both worlds, using robust motion estimation from self-navigation to robustly calibrate PT measurements, thus allowing the latter to be later used for effective motion correction of sequences with arbitrary contrast or sampling schemes. To this end, we propose PT with a distributed motion calibration (DMC) protocol that is short enough that it can be integrated unobtrusively between scans. The flexibility of this approach results in a clinically adoptable solution. Furthermore, the distributed layout promotes capturing the range of poses that occurs during the full examination period without relying on patient cooperation. Using self-navigated sampling for the DMC acquisition ensures robust and efficient calibration, and reliable motion estimates can be obtained at a high temporal resolution (e.g., 0.3 s). Finally, we propose a data-driven calibration refinement during each reconstruction that further improves motion correction tailored to each acquisition. The suggested approach does not rely on sequence modifications and complements the conventional reconstructions with an additional motion-corrected one.

## 2 Theory

### 2.1 AlignedSENSE motion correction

The proposed DMC method is built on alignedSENSE motion correction.^29^ For volumetric encoding using array receiver coils and Cartesian sampling, motion correction is achieved by dividing k-space readouts into temporal segments and allowing each segment *n* to have a distinct motion state (***z_n_***) that can be estimated on top of the reconstructed volumetric image (***x***). The generalized reconstruction with rigid motion correction for parallel imaging can be formulated as an inverse problem in matrix form as follows:

(1) $$\left(\hat{x},\hat{z_{n}}\right)={\text{argmin}}_{x,z_{n}}\underset{n\prime =1:N}{\sum }\|A_{n^{\prime }}FST\left(z_{n^{\prime }}\right)x-y_{n^{\prime }}{\|}_{2}^{2}$$

where ***S*** are sensitivity profiles of the C-element coil receiver array, and ***F*** is the discrete Fourier transform (DFT). A running variable *n*′ is used to loop over all *N* segments. For each segment *n*′, ***T***(***z***_*n*′_) is the rigid motion operator with motion parameters ***z***_*n*′_; ***A***_*n*′_ is the sampling mask; and ***y***_*n*′_ is the measured data for all coil elements. The structure and sizes of the operators in Eq. (1) can be found in Brackenier et al.^33^

Although Eq. (1) can in theory be used for any sampling trajectory, robust performance requires the use of self-navigated sampling schemes, defined as sampling schemes in which the sampling points from each segment *n* (***A***_*n*_) contain the necessary spatial encoding to infer rigid pose parameters. The self-navigated DISORDER sampling, in which each segment collects samples spanning across k-space, is an established option and forms the basis of the proposed MC implementation.

### 2.2 PT calibration

**Figure 1:**
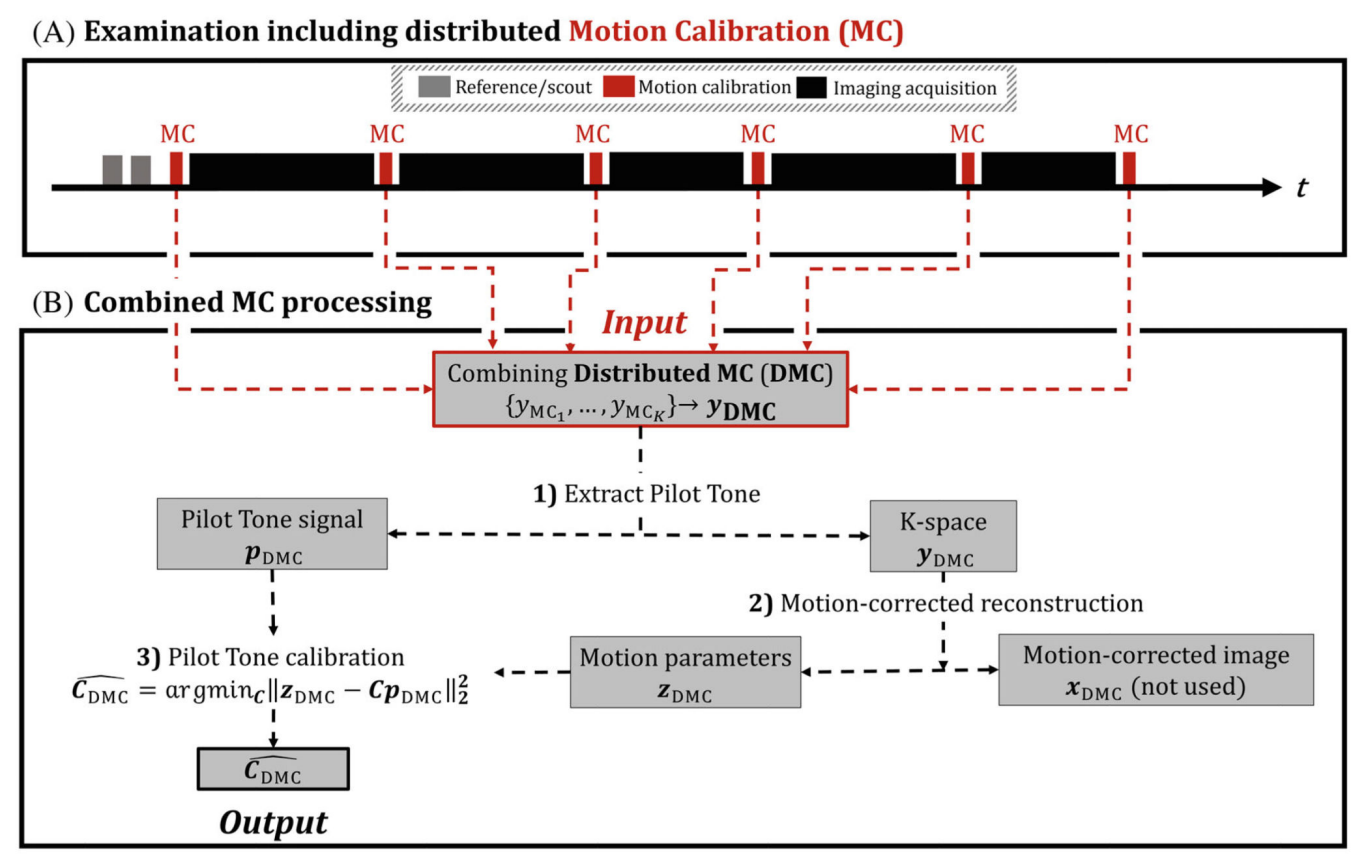
Distributed motion calibration pipeline (DMC). (A) Throughout an examination, multiple short motion calibration (MC) blocks (*red*) are placed. A distributed placement promotes MC acquisitions to capture the range of motion occurring throughout imaging acquisitions (*black*). (B) MC acquisitions are combined and processed together (1) to provide robust motion estimation (2); estimated motion, together with extracted Pilot Tone signals are then used to build a calibration model (3).

**Figure 2:**
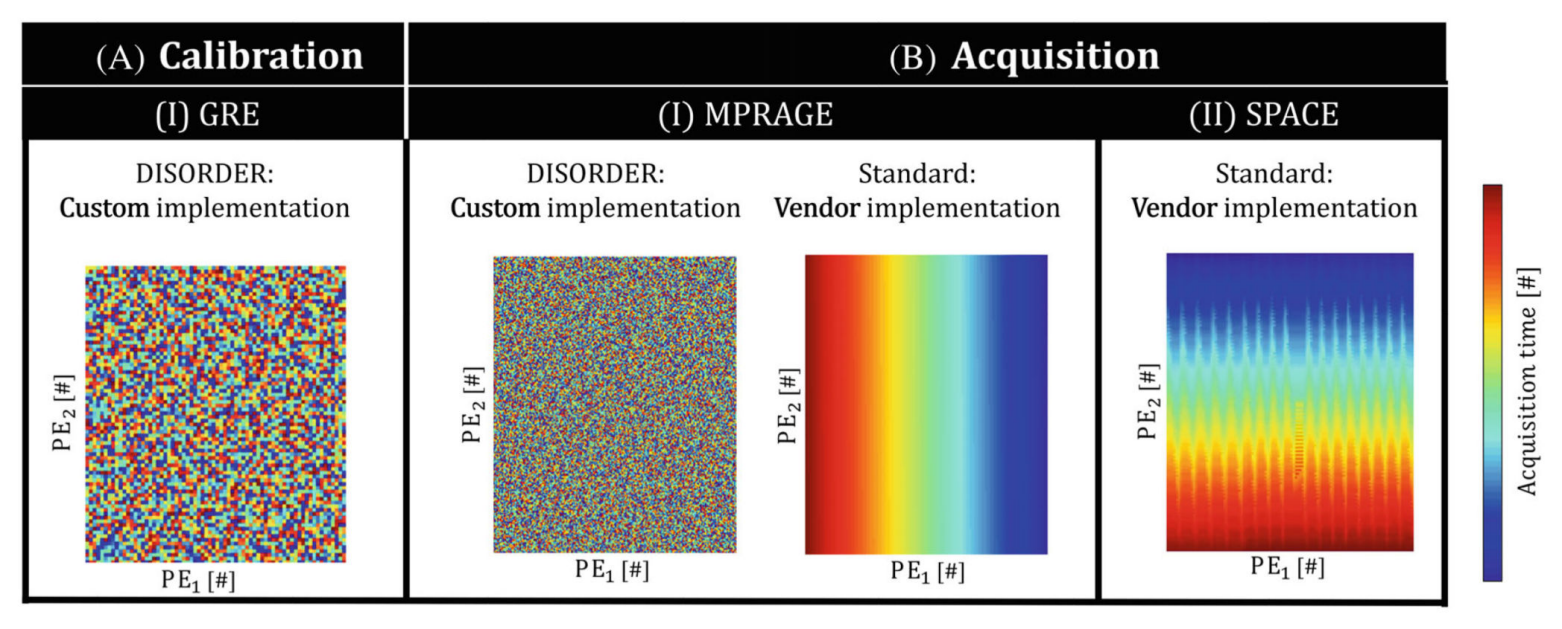
k-Space sampling schemes in the Cartesian phase-encoding plane. Sampling schemes are shown for the calibration (A) and imaging acquisitions (B) used in this work. The custom DISORDER implementation is shown (A.I−II) next to the standard sampling schemes for the MPRAGE and SPACE sequences (B.I−II). For the SPACE sequences, the standard sampling contained an approximately linear sampling with added jittering. GRE, gradient-recalled echo.

The DMC protocol acquires a set of *K* blocks of motion calibration (MC) k-space data ***y***_MC_ during which PT signals are simultaneously acquired. To allow robust motion estimation, the current implementation uses a DISORDER sampling scheme (Figure 2A) where ***y***_MC_ consists of several DISORDER segments (with about 0.3−0.5-s duration). All ***y***_MC_ are distributed between regular acquisitions with at least one at the start and end of the exam (Figure 1). Next, MC acquisitions are combined into ***y***_DMC_ = {MC_1_, …, MC_*K*_}. Motion parameters corresponding to each segment *n* within ***y***_DMC_ $\left(z_{{\text{DMC}}_{n}}\right)$ are obtained using the alignedSENSE framework (Eq. [1]):$y_{\text{DMC}}\to {\hat{x}}_{\text{DMC}},{\hat{z}}_{{\text{DMC}}_{n}}$. The total number of estimated motion parameters (*N*) depends on both the number of MC blocks (*K*) and the number of segments in each block. Because DMC acquisitions are only used for motion estimation, MC acquisitions can be low-resolution and can have arbitrary contrast properties (the images themselves are discarded).

PT signals are acquired during the DMC acquisitions and are extracted for each readout (see Section 3). After averaging PT signals across segment $n\left(p_{{\text{DMC}}_{n}}\right)$, a linear relationship between $z_{{\text{DMC}}_{n}}$ and $p_{{\text{DMC}}_{n}}$ can be assumed when using a head coil receiver array as follows^13,34^:

(2) $$z_{{\text{DMC}}_{n}}=Cp_{{\text{DMC}}_{n}}$$

where $p_{{\text{DMC}}_{n}}$ is a complex vector containing *N*_C_-channel PT signal and concatenated with a 1 to allow for a motion offset, and ***C*** is a 6×(*N*_C_ + 1) matrix with the rows containing the calibration coefficients for each of the six rigid motion parameters. Given the estimated motion parameters $z_{{\text{DMC}}_{n}}$, a calibration matrix $\hat{C_{\text{DMC}}}$ can then be estimated using a least-squares fit:

(3) $$\hat{C_{\text{DMC}}}={\text{argmin}}_{C}\underset{n^{\prime }}{\sum }\|z_{\text{DMC},n\prime }-Cp_{\text{DMC},n^{\prime }}{\|}_{2}^{2}$$

Note that when Eq. (3) is performed with nondistributed data, the notation $\hat{C_{\text{MC}}}$ is adopted.

### 2.3 PT-alignedSENSE: extension of alignedSENSE to incorporate PT calibration

The PT motion model from Eq. (2) (***z***_*n*_ = ***Cp***_*n*_) can be incorporated into the alignedSENSE framework (Eq. [1]), referred to as PT-alignedSENSE. Two variations of the PT-alignedSENSE are explored.

First, the precalibrated model $\hat{C_{\text{DMC}}}$ can be used without further refinement and simply using the PT-predicted motion parameters, referred to as the *PT-enforced* alignedSENSE:

(4) $$(\hat{x})={\text{argmin}}_{x}\underset{n^{\prime }}{\sum }\|A_{n^{\prime }}FST\left(\hat{C_{\text{DMC}}}p_{n^{\prime }}\right)x-y_{n^{\prime }}{\|}_{2}^{2}$$

Second, $\hat{C_{\text{DMC}}}$ can be used to initialize a data-driven calibration refinement that is optimized in combination with image reconstruction, referred to as the *PT-guided* alignedSENSE:

(5) $$(\hat{x},\hat{C})={\text{argmin}}_{x,C}\underset{n^{\prime }}{\sum }\|A_{n^{\prime }}FST\left(Cp_{n^{\prime }}\right)x-y_{n^{\prime }}{\|}_{2}^{2}$$

To make the optimization in Eq. (5) converge for standard sampling schemes (e.g., linear phase encoding), ***C*** is carefully initialized with $\hat{C_{\text{DMC}}}$. However, convergence can be reached without initialization when using PT-alignedSENSE with self-navigated trajectories.^34–36^

Equation (5) can be solved by iteratively updating ***x*** and ***C***:

(6a) $$x^{i+1}={\text{argmin}}_{x}\underset{n\prime }{\sum }\|A_{n\prime }FST\left(C^{i}p_{n\prime }\right)x-y_{n\prime }{\|}_{2}^{2}$$

(6b) $$C^{i+1}={\text{argmin}}_{C}\underset{n^{\prime }}{\sum }\|A_{n^{\prime }}\text{FST}\left(Cp_{n^{\prime }}\right)x^{i+1}-y_{n^{\prime }}{\|}_{2}^{2}$$

Equations (6a) and (6b) use the conjugate gradient and Levenberg−Marquardt (LM) algorithms, respectively. The formulation of the LM update used in Eq. (6b) is described in Appendix A.

## 3 Methods

### 3.1 PT signal generation, extraction, and preprocessing

**Figure 3:**
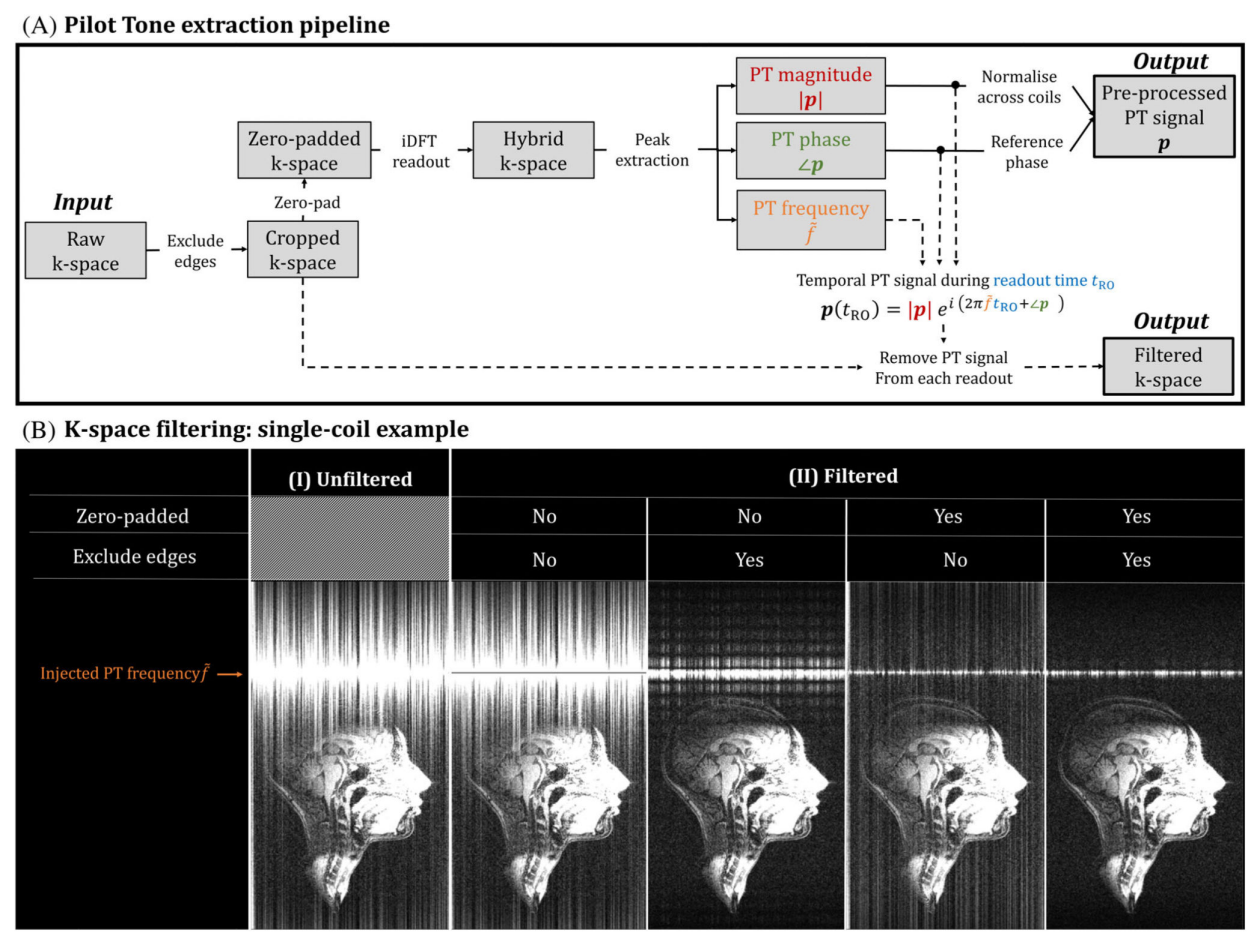
Pilot Tone (PT) extraction pipeline. (A) PT signals are extracted from the raw k-space by a set of sequential steps. First, k-space is cropped, followed by zero-padding to achieve sinc interpolation in the hybrid k-space. The latter is achieved by performing the inverse discrete Fourier transform in the readout dimension. Finally, the PT signal is obtained by extracting the peak of the hybrid k-space. After extraction, PT signals are preprocessed by normalizing the magnitude across the coil dimension and by referencing the phase to an arbitrary channel. (B) PT signals are removed from the raw k-space to avoid leakage artifacts in the reconstructed images.

For all experiments, PT signals were generated with an RF signal generator (APSIN3000; AnaPico, Glattbrugg, Switzerland) and broadcast into the magnet room using a monopole antenna connected to a feedthrough panel at the head end of the scanner room. The frequency of the injected PT signal (*f*_PT_) was set to

(6) $$f_{\text{PT}}=f_{B_{0}}+\text{BW}\left(\frac{{\text{ISO}}_{\text{RO}}}{{\text{FOV}}_{\text{RO}}}+q\right)$$

where $f_{B_{0}}$ is the scanner’s center frequency; BW is the readout (RO) bandwidth; FOV_RO_ is the FOV in the RO dimension; ISO_RO_ the FOV offset in the RO with respect to the isocenter; and *q* is the factor determining where the signal appears (*q*=− 0.5/0.5 corresponding to the lower and upper edge of the non-oversampled FOV, respectively). A factor *q* = 0.45 was used in this work to set the PT superior to the head in the FOV_RO_ to not overlap with any MR signal (Figure 3B). To achieve sufficient PT SNR, the drive level of the generator was set to −10 dBm.

PT signal acquired during the readout at TR (***p***_TR_ with magnitude ∣ ***p***_TR_ ∣ and phase ∠***p***_TR_) is superposed onto the normally acquired k-space readout (***y***_TR_) as follows:

(7) $$y_{{\text{TR}}_{\text{meas}}}=y_{\text{TR}}+\left|p_{\text{TR}}\right|e^{i[2\pi \left({\tilde{f}}_{\text{PT}}{\text{t}}_{\text{RO}}+∠p_{\text{TR}}\right]}$$

where ${\tilde{f}}_{\text{PT}}=f_{\text{PT}}-f_{B_{0}}$ is the apparent frequency after demodulation. The values of |***p***_TR_|, ∠***p***_TR_, and ${\tilde{f}}_{\text{PT}}$ can be extracted from each $y_{{\text{TR}}_{\text{meas}}}$ by taking the inverse DFT along the RO (hybrid k-space) and detecting the peak.^10^ Because both *f*_PT_ and $f_{B_{0}}$ can shift throughout acquisitions (e.g., due to temperature related drift), ${\tilde{f}}_{\text{PT}}$ is estimated for every TR independently (Figure 3A). To capture subtle changes in ${\tilde{f}}_{\text{PT}}$, the spectral resolution is increased by zero-padding ***y***_TR_ before taking the inverse DFT and achieving a sinc-interpolated hybrid k-space.^37^ The size of zero-padding is set to 30 times the original size. Additionally, 5% from both edges of ***y***_TR_ are removed (cropped), as $y_{{\text{TR}}_{\text{meas}}}$ shows jumps at the start and end of the RO, hypothesized to be caused by opening and closing the ADC (see Figure S1). After detecting the PT signal, it is removed from the raw k-space used for subsequent image reconstruction by subtracting the second term of Eq. (7). Without filtering, PT signals can leak onto the remaining FOV and overlap with the MR signal. This is a well-known DFT property when dealing with incommensurate sampling.^37^

The extracted PT signal, ***p***_TR_, undergoes a set of preprocessing steps. The magnitude ∣ ***p***_TR_ ∣ is modified to have a unitary norm across coil elements, accounting for global drifts in ∣ ***p***_TR_ ∣ (e.g., RF generator, respiration), as we are only interested in differential changes between coil elements. For the same reason, the phase ∠***p***_TR_ was referenced to the first channel. Next, real and imaginary components of the PT signal are concatenated across the channel dimension to make Eq. (2) noncomplex and simplify gradient expressions for the LM update in Eq. (6b). Finally, a dimensionality reduction using principal component analysis (PCA) was applied to avoid overfitting in Eq. (3) and to reduce the number of parameters to estimate in Eq. (6b), $p_{n}\to p_{n}^{\text{PCA}}$, with an associated change in calibration matrix ***C***→ ***C*** ^PCA^. The number of principal components is truncated to *N*_PC_, and this defines the shape of both $p_{n}^{\text{PCA}}$ and $C^{\text{PCA}}(p^{\text{PCA}}\in {\mathbb{R}}^{\left(N_{\text{PC}}+1\right)\times 1}$ and $C^{\text{PCA}}\in {\mathbb{R}}^{6\times \left(N_{\text{PC}}+1\right)}$).

### 3.2 In vivo data acquisition

Volumetric acquisitions were acquired on 12 adult healthy volunteer (HV) subjects on a 7T scanner (MAGNETOM Terra; Siemens Healthcare, Erlangen, Germany) with a 1Tx adult head coil (Nova Medical) using the inbuilt *N*_C_ = 32-element receiver coil array. Volunteers gave written consent, and data were acquired as part of an ethically approved study. For each HV, three MPRAGE acquisitions were acquired: one with a DISORDER sampling (segment duration TA_segment_ = 0.76 s) and the other two using a standard linear sampling scheme. For the last 8 HVs, two standard SPACE (sampling perfection with application optimized contrast using different flip angle evolution) sequences were additionally acquired, each with the standard sampling scheme provided by the vendor. The HVs were instructed to remain still for each of the first standard MPRAGE and SPACE acquisitions, then instructed to move by drifting slowly throughout the scan for the second of each acquisition. These are later referred to as “low motion” and “increased motion” acquisitions.

Sampling patterns for all acquisitions are shown in Figure 2. Note that the SPACE sampling scheme contains a jittered ordering, making it semi-self-navigated. Additional MPRAGE sequence parameters were 1 × 1 × 1 mm^3^, TE/TR_short_/TR_long_ = 1.48/2.96/3000 ms, TI = 1400 ms, flip angle (FA) = 8°, FOV = 240 × 210 × 256 mm^3^, no acceleration, and acquisition time (TA) = 10 min 35 s. Additional SPACE sequence parameters were 1 × 1 × 1 mm^3^, TE/TR_short_/TR_long_ = 385/3.07/3000 ms, variable flip angle with FA_0_ = 120°, FOV = 240 × 186 × 240 mm^3^, acceleration *R* = 1.5 × 1.5, and TA = 4 min 24 s.

Acquired calibration data consisted of six individual fully sampled MC acquisitions placed throughout the examination (Figure 1A). Note that the HVs were not instructed to perform any movement during each MC acquisition. Additional MC sequence parameters were gradient-recalled echo, 3 × 3 × 3 mm^3^ resolution, FOV = 240 × 212 × 240 mm^3^ (inferior−superior [IS]/anterior−posterior [AP]/left−right [LR]), TE/TR = 1.93/3.8 ms, FA = 8°, and scan duration TA = 22 s. A DISORDER segment duration TA_segment_ = 0.33 s ensures motion estimates at high temporal resolution.

For all acquisitions, an IS RO was used to exclude the neck region from rigid motion estimation^26^ and a bandwidth (BW) of 870 Hz/mm was used to suppress distortion. The BW and ISO_RO_ were fixed for all acquisitions to have a fixed *f*_PT_. For each HV, coil array sensitivity profiles were estimated from a separate low-resolution (4 × 4 × 4 mm^3^) scan using a custom implementation of the ESPIRiT algorithm.^38^

To mimic the proposed *distributed* MC acquisition, one-sixth (17%) of the k-space from each MC acquisition was combined to construct a single fully sampled k-space data set. The extraction from each MC corresponds to an approximate 4-s acquisition time, which is short enough to be unobtrusively inserted between standard acquisitions. The *nondistributed* alternative with equivalent TA was obtained by choosing a single fully sampled MC acquisition out of all six MC acquisitions. To explore the effect of total acquisition data on calibration adequacy, DMC data were constructed by extracting different fractions (one sixth, three sixths, and six sixths) from each MC acquisition, corresponding to an equivalent total TA of 22/66/132 s. DMC data are labeled based on the equivalent number of fully sampled MC acquisitions it contains: DMC_1_, DMC_3_, and DMC_6_ for the aforementioned extraction fractions.

### 3.3 In vivo experiments

For each calibration data set (DMC_1_/DMC_3_/DMC_6_ or each individual MC acquisition), a precalibrated $\hat{C}$ was constructed following Section 2.2. The value of $\hat{C}$ is validated using the DISORDER MPRAGE acquisition. The latter, having self-navigation, is assumed to achieve reliable motion parameters after motion correction. Therefore, these motion parameters are used as the reference values (***z***_ref_). Next, these motion parameters are predicted using the PT signals ***p***_ref_ extracted from the DISORDER MPRAGE: $\hat{z_{\text{ref}}}=\hat{C}p_{\text{ref}}$. Experiment 1 compares the performance between the distributed and no-distributed processing of MC data consisting of a single fully sampled MC acquisition (DMC_1_ and a single MC, respectively). To exhaustively test the nondistributed calibration, validation is repeated for each individual MC acquisition. Experiment 2 compares the DMC processing for the range of extraction fractions (DMC_1,3,6_). For Experiments 1 and 2, validations are performed for a range of *N*_PC_ and across all HVs. Mean errors are discarded, as offsets in motion traces are irrelevant for image reconstruction: They simply shift the baseline pose of the reconstructed image. Therefore, the SD (σ) of the error in motion parameters is used as the validation metric. Due to a signal drop in the PT signal after MC_1_ in both HV 3 and 11 (see Section 5), MC_1_ was excluded when performing Eq. (3).

Experiment 3 reconstructs all MPRAGE and SPACE acquisitions using different methods:

(i) uncorrected, corresponding to SENSE^39^ (*noniterative*);
(ii) conventional motion-corrected without leveraging PT, corresponding to alignedSENSE (*iterative*);
(iii) “PT-enforced motion-corrected” (*noniterative*); and
(iv) “PT-guided motion-corrected” (*iterative*).

Reconstructions (iii) and (iv) are performed using $\hat{C}$ from both the *nondistributed* (MC_1_) and *distributed* (DMC_1_) calibration extraction. Additionally, reconstructions are performed with DMC_3_ and DMC_6_ to investigate the effect of using more calibration data.

For MPRAGE reconstructions, the motion-corrected DISORDER MPRAGE is considered as the reference reconstruction ***x***_ref_. Standard MPRAGE reconstructions are registered to ***x***_ref_, and errors are evaluated by computing the signal to residual ratio (SRR): $SRR=|\bar{\hat{x}}{|}^{2}/|\bar{\hat{x}-x_{\text{ref}}}{|}^{2}$. For the SPACE reconstructions, no reference is available, and the image quality is assessed using the normalized gradient squared (NGS)^40^: $\text{NGS}=\sum_{k=1:3}\left\||D_{k}\hat{x}|/\right\|D_{k}\hat{x}{{\|}_{1}\|}_{2}^{2}$, where ***D***_*k*_ represents the finite difference along dimension *k*. Brain masks are extracted using BET (Brain Extraction Tool) and are used when computing the SRR and NGS.^41^ Both image-quality metrics are referenced with respect to the uncorrected reconstruction to account for a variety in baseline image quality across subjects: (NGS − NGS_uncorrected_)/NGS_uncorrected_ and (SRR − SRR_uncorrected_)/SRR_uncorrected_.

For computational efficiency, motion was estimated at half the resolution, and coil profiles are compressed to 17 virtual coils.^42^ The implementation builds on the one presented in Cordero-Grande et al.^26^ Reconstructions are performed offline on a 20(40) × Intel Xeon Silver 4210 2.20 GHz CPU, 251 GB RAM, 32GB NVIDIA Tesla V100 GPU. The maximum computation time for a 1 × 1 × 1 mm^3^ motion-corrected reconstruction was 23 min.

### 3.4 Statistical analysis

Statistical analyses were conducted using a significance level of *p* < 0.05. For each test, the normality assumption for the relevant data was assessed using the Kolmogorov−Smirnov test. In all instances, the null hypothesis was rejected, resulting in the application of nonparametric tests. The unpaired Mann−Whitney U-test was used to assess the enhanced performance of distributed versus nondistributed MC acquisition in Experiment 1. All motion correction methods from Experiment 3 were subjected to a group-wise, paired Friedman test, assessing the null hypothesis that all corrections originate from the same distribution. If the null hypothesis was rejected, a subsequent comparison was executed between methods of interest (see Section 4): The Wilcoxon signed rank test was used to assess the enhanced performance of individual methods. Finally, A Wilcoxon signed rank test was performed to test whether PT-informed motion correction deteriorated performance compared with the conventional motion correction. Statistical tests were performed using *MATLAB*’s built-in functions.

## 4 Results

### 4.1 k-Space filtering

Figure 3B shows the effect of the k-space filtering for a single coil element. Without filtering (Figure 3B.I), the PT signal strongly overlaps with the MR data. Figure 3B.II shows different filtering options. Without considering edge exclusion or zero-padding, only a single frequency (*orange arrow*) is extracted, which does not remove the leakage overlapping the head. Although this effect can be minimized by lowering the drive level of the PT generator, this would lead to noisier PT signals. Applying both filtering options individually (*middle*) results in clear improvements, with the combined application (*right*) resulting in optimal performance without noticeable signal overlap. After filtering, the residual PT signal superior to the head is set to zero to suppress residual PT signals. The estimated PT frequencies $\tilde{f}$ (results not shown) vary with the sequence layout.

### 4.2 In vivo experiments

**Figure 4:**
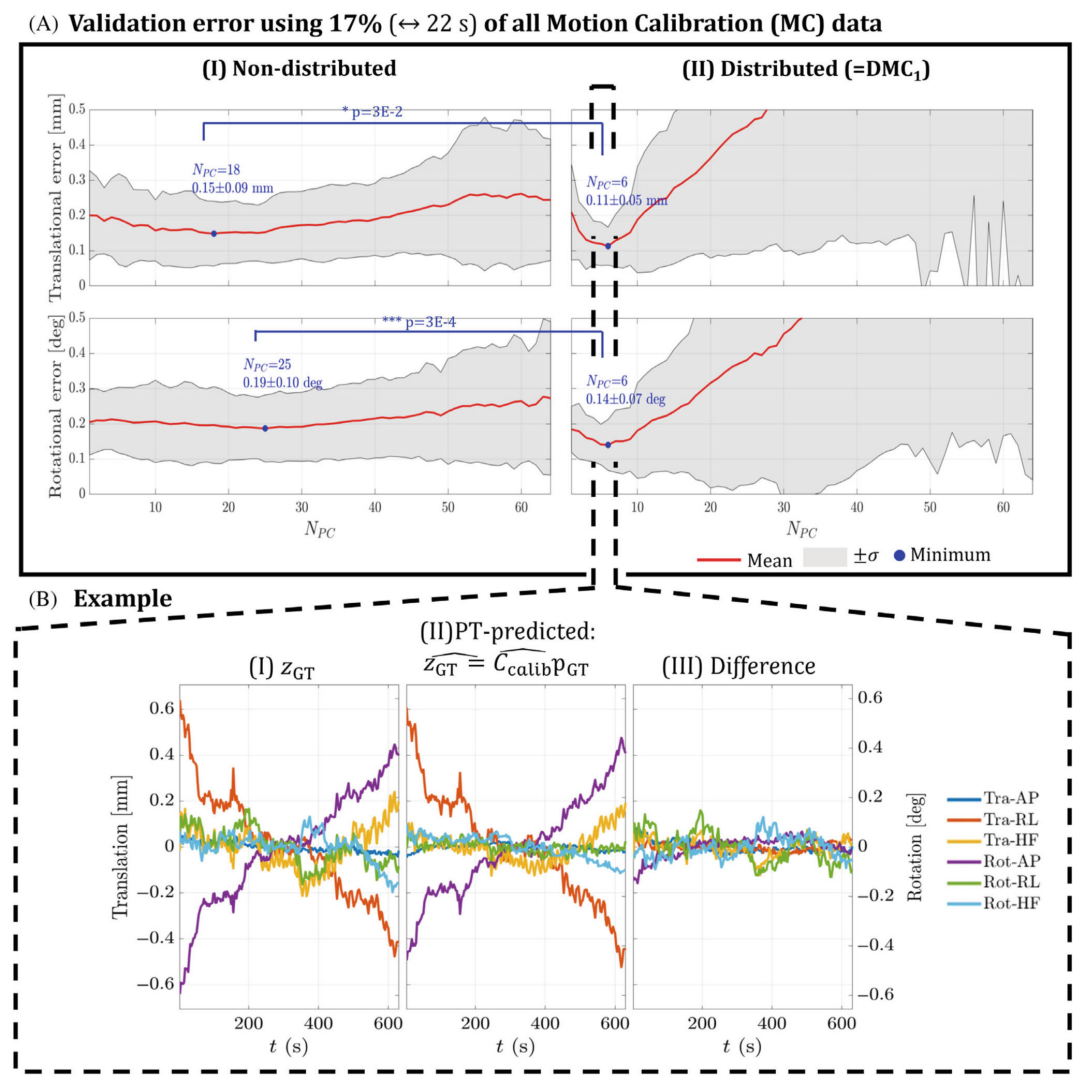
Experiment 1. (A) Validation errors in translation and rotation are shown for a range of principal components (*N*_PC_) when using both nondistributed (A.I) and distributed (A.II) calibration data. The comparison is shown for motion calibration (MC) data corresponding to 17% of the total calibration acquisition. For the optimal *N*_PC_ (*blue*), lower residuals are observed for the distributed case (DMC_1_). In the latter, strong dependence on *N*_PC_ is obtained. (B) An example of the motion prediction for a single healthy volunteer.

Results for Experiment 1 are shown in Figure 4. Figure 4A shows the validation error across all HVs when using 17% of all acquired MC data. Validation errors are shown for the range of possible number of *N*_PC_ for both translation (*top*) and rotation (*bottom*). The distributed approach (DMC_1_) shows strong sensitivity to *N*_PC_, with a global optimum (*blue*) at *N*_PC_ = 6. This contrasts with the nondistributed case, in which performance is much less sensitive to *N*_PC_. For the optimal *N*_PC_, the distributed data extraction in Figure 4A.II results in significantly lower residuals compared with a nondistributed approach in Figure 4A.I: 0.11 ± 0.05 mm ↔ 0.15 ± 0.09 mm (*p* = 3*E*^−2^) and 0.14 ± 0.07 deg ↔ 0.19 ± 0.10 deg (*p* = 3*E*^−4^) for translation and rotation, respectively. An example of the reference and predicted motion using the calibration model is shown in Figure 4B.I−II, with differences (< 0.2 mm/deg) that are small compared with the movements (Figure 4B.III).

Results for Experiment2 are shown in Figure S2, which presents the validation error for DMC data for a range of extraction fractions (DMC_1-6_). Using more calibration data results in lower validation errors, especially for higher *N*_PC_. Additionally, the optimal *N*_PC_ increases and the associated optimum becomes less sensitive to *N*_PC_, with optima at 6/9/19 for the different DMC_1_/DMC_2_/DMC_3_, respectively. The effect of choosing different *N*_PC_ on image reconstructions is shown in Figure S3; nonoptimal choices resulted in decreased performance. As a result, these optimal values are adopted for the reconstructions in Experiment 3.

**Figure 5:**
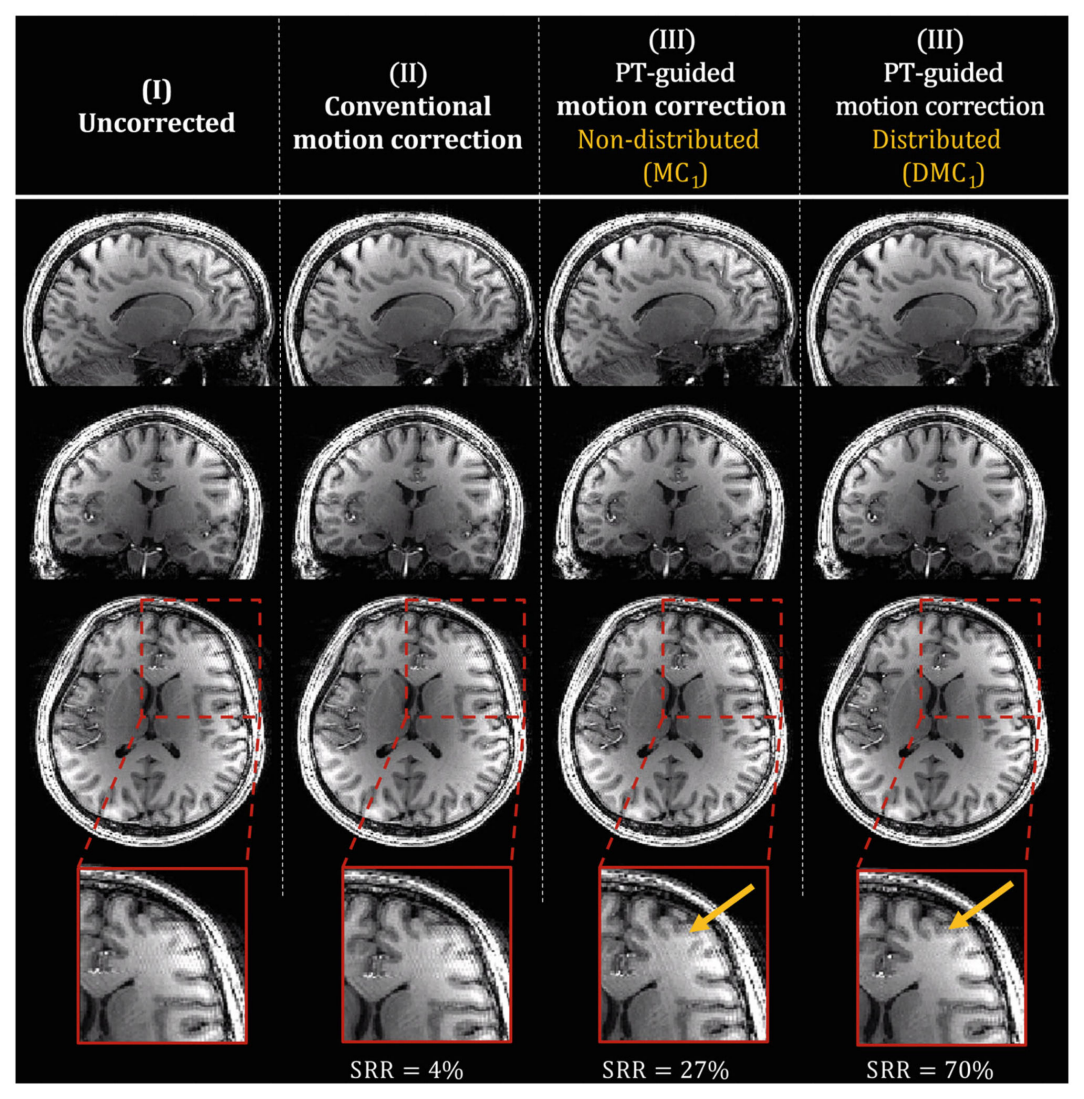
Reconstruction for the standard MPRAGE acquisition (linear phase-encoding sampling scheme) from Healthy Volunteer 7. Reconstructions are shown for no correction (I), conventional motion correction (II), and the Pilot Tone (PT)−guided motion correction (III−IV). PT-guided motion correction is performed when using nondistributed (III) and distributed (IV) calibration data worth 22 s of acquisition time (MC_1_ and DMC_1_, respectively). Signal to residual ratio improvements with respect to the uncorrected case are shown for each reconstruction and show improved performance when using DMC_1_. DMC, distributed motion calibration; MC, motion calibration.

**Figure 6:**
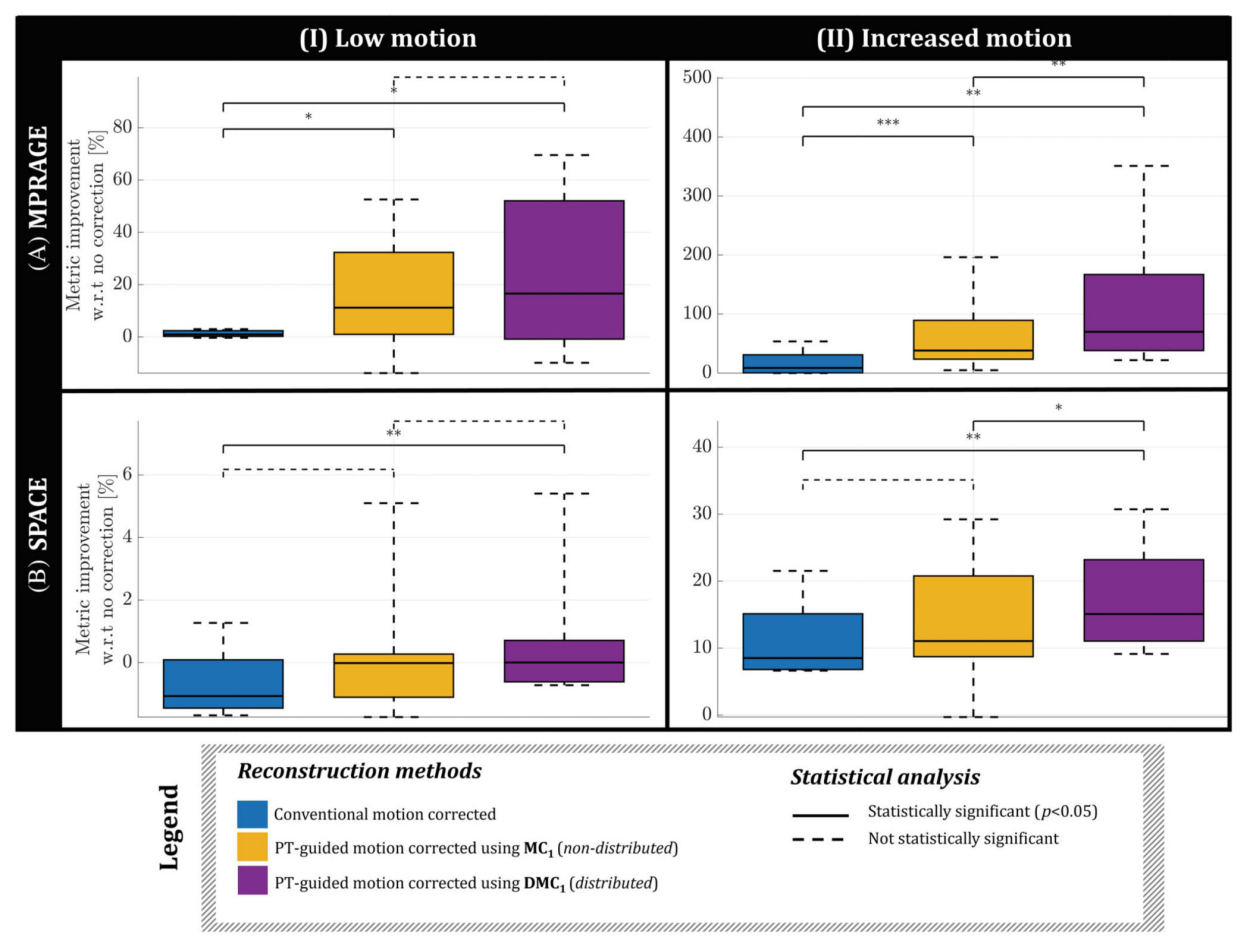
Image quality metric across healthy volunteers (HVs) shown for the motion-corrected reconstructions displayed in Figure 5. Boxplots (across HVs) are shown for both MPRAGE (A) and SPACE (B) sequences, and for the low-motion (I) and increased-motion (II) experiment. The signal to residual ratio and normalized gradient squared metrics are used for the MPRAGE and SPACE acquisitions, respectively. Significance levels are shown for each Pilot Tone (PT)−guided motion correction compared with the conventional motion correction. Note that the statistical tests were pairwise, explaining significant differences found even when the boxplots (showing variability across subjects) are largely overlapping. DMC, distributed motion calibration; MC, motion calibration. *, **, *** corresponds to *p* < 0.05, 0.005, 0.0005.

Results for Experiment 3 are shown in the remaining figures. Figure 5 compares the PT-guided motion correction using distributed and nondistributed calibration data (calibration TA = 22 s). Reconstructions of the standard MPRAGE acquisition from HV 7 are shown without correction (Figure 5A), with conventional motion correction (Figure 5B) and with the PT-guided motion correction using MC_1_ (Figure 5C) and DMC_1_ (Figure 5D) for precalibration. Even when using 22 s worth of calibration data, PT-guided motion corrections outperformed the conventional motion correction (27% and 70% vs. 4% SRR improvement), with the distributed approach achieving the highest SRR improvement and artifact removal (*arrows*). Boxplots showing metrics across all HVs are shown in Figure 6 for both sequences (MPRAGE/SPACE) and motion experiments (low/increased motion). Consistent with observations in Figure 5, a distributed calibration approach (*purple*) outperforms the nondistributed approach (*yellow*); median metric improvements are higher, and significant improvement (*p* < 0.05) with respect to the conventional motion correction is obtained for all cases, whereas the nondistributed approach only achieves this for MPRAGE data. Furthermore, in the increased-motion experiment, the distributed calibration was significantly better than the nondistributed calibration. It is noteworthy that the conventional motion correction deteriorated image quality for the SPACE low-motion experiment, which was not the case for PT-guided motion corrections. In all MPRAGE and SPACE experiments, PT-guided motion correction did not show significant deterioration, indicating noninferiority.

**Figure 7:**
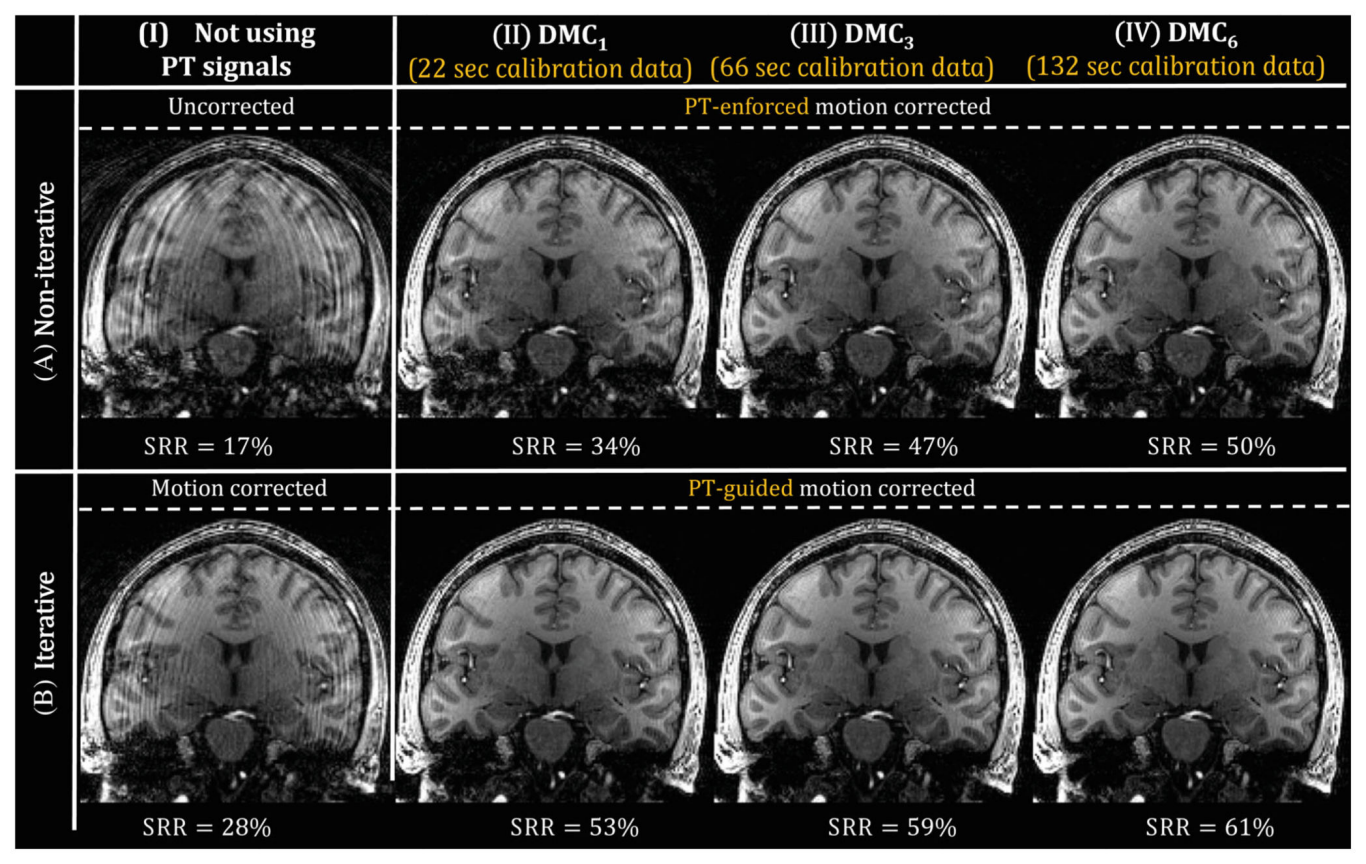
Reconstructed images for Healthy Volunteer 2 using different reconstruction methods, categorized into noniterative (A) and iterative (B) reconstructions. The proposed method is evaluated for a range of distributed motion calibration (DMC) extractions (II−IV) and are compared with the uncorrected (A.I) and conventional (B.II) motion correction. The proposed correction is performed using the noniterative (Pilot Tone [PT]−enforced) or iterative motion correction (PT-guided). The signal to residual ratio (SRR) improvements are shown for every reconstruction. PT-informed motion correction outperforms the conventional motion correction, with best performance when using the scan-specific calibration refinement (PT-guided).

Figure 7 shows reconstructions of the standard MPRAGE with increased motion levels for HV 2. Reconstructions are shown for noniterative (Figure 7A) and iterative (Figure 7B) approaches and different amounts of calibration data used (Columns I−IV, where I does not use any PT data). Using the precalibrated matrix $\hat{C_{\text{DMC}}}$ without refinement (PT-*enforced* alignedSENSE in Figure 5A.II−IV) outperforms the conventional motion correction (Figure 5B.I), with more calibration data resulting in improved performance (34/47/50% SRR improvement). The proposed scan-specific calibration refinement (PT-*guided* alignedSENSE) further improves the performance (Figure 5B.II−IV with 53/59/61% SRR improvement). Statistical tests across HVs in Figure S4 confirm that the calibration refinement in the PT-guided motion correction significantly improves the PT-enforced motion correction. Similarly, results for all DMC fractions across HVs are shown in Figure S5 and confirm that using more calibration data improves overall performance of PT-alignedSENSE motion correction. A detailed overview of all the reconstruction methods’ metrics for individual HVs is shown in Figure S6. Individual cases exist (e.g., HV 10 in the low-motion experiment) in whom the PT-guided motion correction deteriorates image quality compared with no correction. Additionally, cases exist in whom increased amounts of DMC data do not further improve performance (e.g., HV 11).

**Figure 8:**
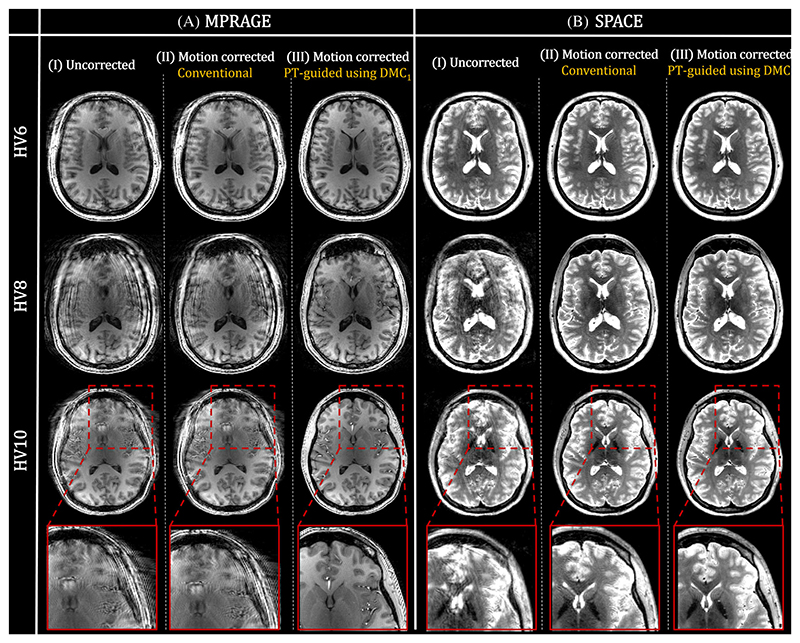
Reconstructed images for the standard MPRAGE (A) and SPACE (B) sequences shown for 3 different healthy volunteers (HVs) across the rows. The uncorrected (I), conventional motion-corrected (II), and the proposed Pilot Tone (PT)−guided motion-corrected (using DMC_1_) (III) images are shown for every scan. Improved or similar reconstructions are obtained when applying conventional motion correction, with further improvements when using the proposed motion correction. DMC, distributed motion calibration.

Finally, Figure 8 shows the reconstructed MPRAGE and SPACE images for the increased motion experiment in 3 subjects when using the PT-guided motion correction with DMC_1_. Consistent with image-quality metrics, conventional motion correction cannot improve MPRAGE motion artifacts much (Figure 8A.II), whereas the PT-guided motion correction removes most artifacts (Figure 8A.III). For the SPACE sequence (Figure 8B), most motion artifacts are removed using the conventional motion correction, although additional improvements from the proposed PT-guided method are apparent.

## 5 Discussion

We have presented an approach to perform motion-corrected brain MRI leveraging externally generated PT signals. PT signals are largely independent of the acquisition setup and image contrast, resulting in versatile motion correction, applicable to sequences that may otherwise be difficult to correct. Our method does not modify the sequence and provides a motion-corrected reconstruction *on top of the original reconstruction*. PT signals do not directly provide estimates of motion/head position; instead, they must be calibrated in situ. For this purpose, we propose an efficient DMC protocol placing short blocks of dedicated acquisitions throughout the examination. Distributing these calibration acquisitions throughout the examination promotes capturing the full range of poses occurring without relying on patient cooperation. Each calibration acquisition can be short enough (~4 s) to be easily inserted between scans. Using self-navigated sampling schemes for the DMC acquisitions results in motion estimates at high-temporal resolution, therefore achieving highly efficient PT calibration. Precalibrated models are subsequently used to initialize a scan-specific calibration refinement to motion-correct any other sequence. We show successful in vivo application to standard SPACE and MPRAGE sequences; we confirm that a distributed calibration approach outperforms a nondistributed approach (using equivalent acquisition times) and show that a scan-specific calibration refinement leads to improved performance. Improved motion correction is achieved even when only six of these short approximate 4-s calibration acquisitions have been used, corresponding to DMC data worth 22 s of total acquisition time.

Although PT signals can be injected without directly interfering with the target acquired data, care is needed during data processing to avoid leakage caused by incommensurate sampling. An effective extraction and k-space filtering strategy was presented (Figure 3) that allows PT to be used at the drive levels required to achieve sufficient PT SNR. Estimated PT frequencies vary with sequence layout and are partially caused by eddy-current compensations applied to the raw data: PT signals do not need eddy-current compensations but are still processed through the same data pipeline. This can induce a shift in the demodulated PT frequency.

Experiment 1 in Figure 4 compared the errors in estimation of motion parameters using distributed and nondistributed calibration acquisitions, demonstrating that the former outperforms the latter (*p* = 0.03) when using 22 s worth of calibration data. This figure examines distributions over permutations of using different MC data for calibration. It is the case that some single MC acquisitions can give lower validation errors than a single DMC_1_ in this experimental setup (results not shown). This is expected if, for example, the calibration scan happens to occur very close in time to the scan to be corrected. However, the goal is to have a model applicable to an entire examination.

As shown in Figure 4A.II, the amount of dimensionality reduction using PCA has a strong effect on the calibration performance, and the importance of using the optimal *N*_PC_ was confirmed during image reconstruction (Figure S3). The nondistributed calibration protocol in Figure 4A.I is not very sensitive to *N*_PC_. In the DMC case, this could have to do with temporally discontinuous k-space data collection being more prone to acquisition inconsistencies not accounted for in the signal model. For example, $B_{0},B_{1}^{+}$, and $B_{1}^{-}$ vary with pose^43–45^ and cause deteriorated motion estimation for large ranges of head poses.^33,46^ This is more likely to occur across the entirety of prolonged examinations. Using more calibration data in Experiment 2 (Figure S2) improves the accuracy and reduces the sensitivity to the dimensionality reduction. Moreover, at increased amounts of DMC data, the optimal *N*_PC_ increases. This could be caused by having a more overdetermined optimization problem, resulting in higher-quality motion estimates that are less affected by the aforementioned model imperfections, either during reconstruction (Eq. [5]) or calibration (Eq. [3]). This demonstrates the flexibility of the proposed method. Acquiring more calibration data, when the scanning workflow allows it, stabilizes the overall performance and leads to improved results.

For Experiment 3, which compared reconstruction methods across volunteers, Figure 5 visually and quantitatively shows improved reconstructions for the PT-guided motion correction using a *distributed* versus *nondistributed* calibration acquisition. Furthermore, results in Figure 6 show improved performance of the PT-guided motion correction compared with the conventional reconstructions and show the applicability across multiple contrasts and sampling schemes. Statistical tests confirm this improved performance. Results in Figure 7 show that using larger amounts of DMC data and a data-driven calibration refinement (PT-guided) can achieve better performance compared with the PT-enforced motion correction, as confirmed statistically (see Figures S4 and S5). Figure 8 visually shows successful application of the PT-alignedSENSE motion correction in both MPRAGE and SPACE sequences using standard (i.e., vendor implemented) k-space sampling schemes. The improvements are smaller for the SPACE sequence due to the semi-self-navigated sampling scheme used, resulting in decent motion correction when only considering acquired imaging k-space data. Statistical tests show that PT-alignedSENSE motion-correction variations are not inferior to either no-correction or an established motion-correction method (alignedSENSE).

When looking at individual performance in Figure S6, we identified a few cases in whom PT-alignedSENSE does not deliver as expected. We hypothesize that this could be due to signal fluctuations observed in the PT data in some instances (examples shown in Figure S7) that are unlikely to be induced by rigid head motion. The source of these signal contaminations is not known; they were not removed by preprocessing, so they directly affected the predicted motion states and motion correction. Further investigation is needed to identify the source of these artifacts and to find potential compensation schemes.

As a continuation of this work, further investigation is needed to verify that the approach could be readily generalized to a wider range of sequences, including with different BW or gradient trajectories. Additionally, although a linear model has been used to relate PT signals with motion (as also used by others^14,34,36^), there is no physical reason why this relationship should be expected to be linear. Further investigation into nonlinear models may improve accuracy, particularly in cases of larger motion.

Although the proposed calibration method and PT signals provide accurate and sequence-agnostic motion parameters, they might not always directly result in optimal image reconstructions. For example, retrospective correction of 3D motion in a 2D acquisition might suffer from spin history effects. Additionally, other imaging nonidealities (e.g., ${\text{B}}_{0},B_{1}^{+}$, and $B_{1}^{-}$ variations) can lower the final image quality. As a result, strategies to limit these effects should be incorporated into the proposed PT-informed motion correction to maximize the final image quality.

This paper focused on the use of distributed motion calibration scans as a means to calibrate PT signals. The same type of approach could be considered for other motion measurement systems,^2,6–13^ which also may need calibration. A straightforward example is the Beat Pilot Tone method, which could provide increased motion sensitivity using the exact same acquisition pipeline.^47^

Exploring PT signals for higher-temporal-resolution motion correction is another promising extension that has been proposed in previous work^35,36^; instead of averaging the signal per segment, transient motion within a segment can be resolved as PT measurements are available per TR. Finally, the proposed DMC protocol needs to be implemented so that it automatically acquires the short blocks of calibration data (~4 s) during dead time or dedicated time slots. In the case of the latter, a fixed amount of calibration data could be acquired upfront to ensure enough calibration data when the examination is cut short. Similarly, real-time PT variation can be used to guide the timings of the MC acquisitions: When no PT signal variation (hence head motion) is detected, MC acquisitions could be relocated to time points where more motion is detected. Similarly, when the dimensions spanned by the MC PT signals (in the “PT signal space” before calibration) don’t match the dimensions spanned by the imaging PT signals, a feedback system could be useful and could indicate the lack of adequate calibration data. Other implementation considerations include the resolution (here, arbitrarily chosen 3-mm isotropic), the self-navigated trajectory, as well as non-steady-state behavior. Regarding the latter, potentially registered contrast agents might alter the contrast of certain MC acquisitions. Moreover, when using short blocks of MC data, the transient response (0.9 s for the MC gradient-recalled echo used in this work) must be considered. Ideally, a dummy acquisition should be added at the start of each MC block, resulting in only a minimally increased scan time. Once the workflow is in place, prospective motion correction can also be explored.

## 6 Conclusion

We have developed a framework to perform motion correction for brain MRI leveraging PT signals. This work builds on current state-of-the-art methods by introducing an efficient and clinically adoptable calibration protocol that acquires short blocks of calibration data (~4 s) across the examination that can easily be integrated into the dead-time between scans. The proposed method is independent of the acquisition setup as well as image contrast, resulting in versatile motion correction applicable to standard sequences.

## Supplementary Material

Supporting Information

Appendix
