# Supplementary material for: Sequence-agnostic motion-correction leveraging efficiently calibrated Pilot Tone signals
Source: Magn Reson Med. Author manuscript; Available in PMC 2025 Feb 24. (PMC7617263; doi:10.1002/mrm.30161)

## Supporting Information:

**Figure S1:** Example of the “jumps” at the beginning and end of the acquired k-space readouts. The first 100 readouts (TR) are shown for coil 3 (of the same subject shown in Figure 3). Magnitude (top) and phase (bottom) of the measured k-space ($\boldsymbol{y}_{TR_{meas}}$) are shown. The jumps in the magnitude are indicated (red) and have a width of ~3 redout samples. The potential effect of these jumps on the phase is not visible due to the strong PT-induced phase rolls throughout the readout.


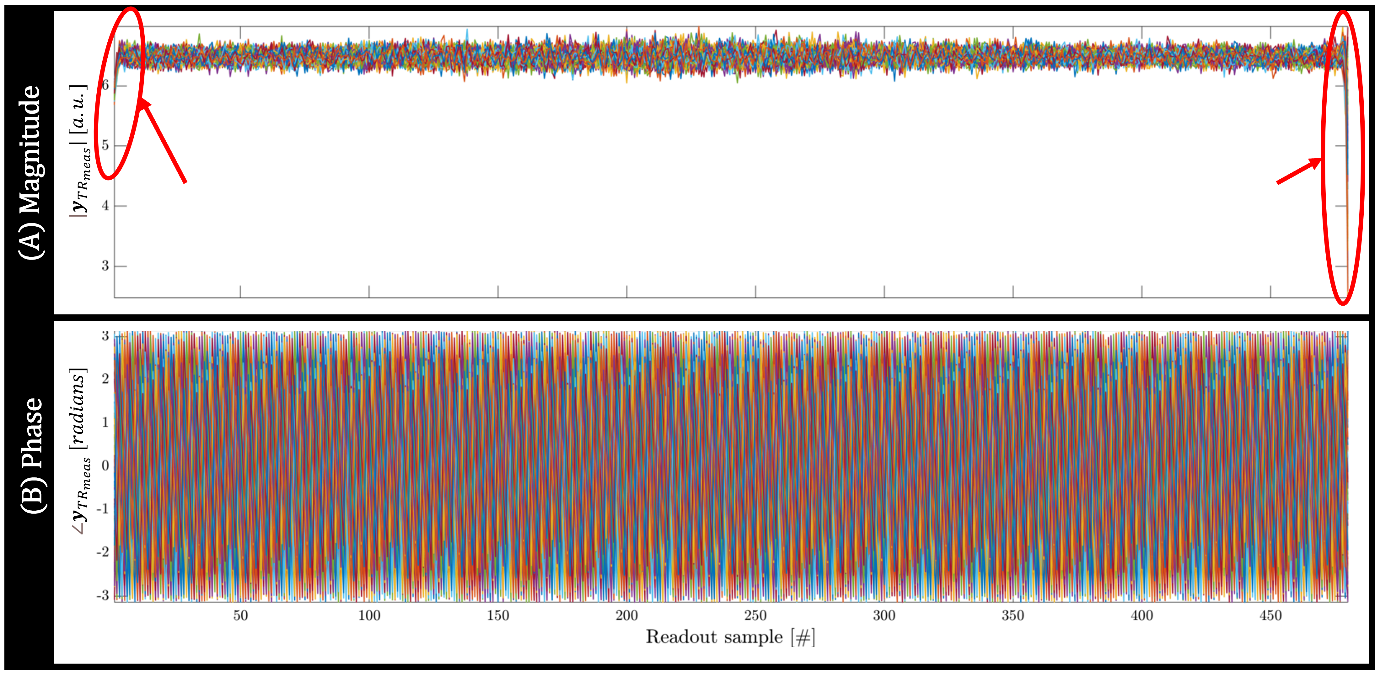


**Figure S2:** Experiment 2: Validation errors in translation (A) and rotation (B) are shown for a range of DMC extractions (I-VI). For increased amount of calibration data (lower rows), the optimal N_PC_ (blue) increases, with lower errors for both translation and rotation. Additionally, the strong dependency on N_PC_ reduces when increasing the total amount of calibration data.


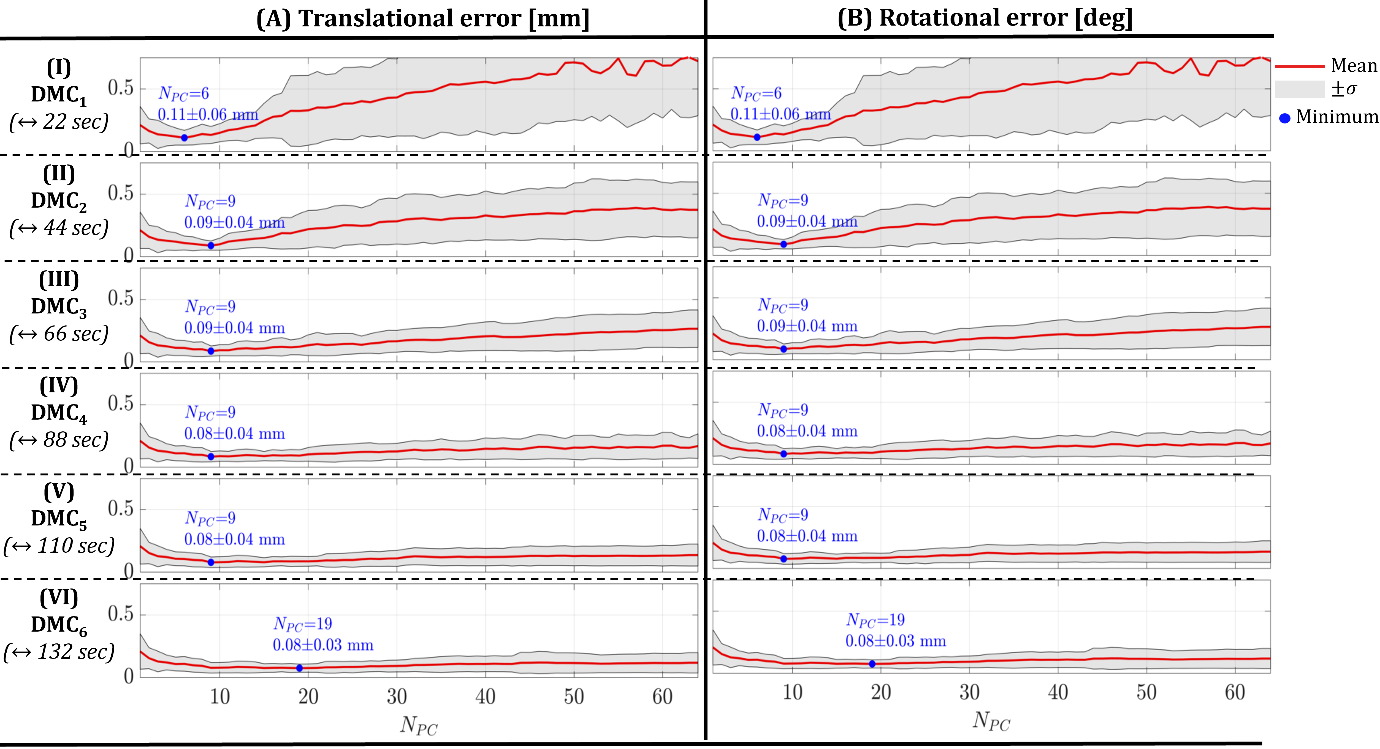


**Figure S3:** PT-guided motion corrections for the standard MPRAGE in healthy volunteer 2 using DMC_1_. Reconstructions are shown for a range of principal components (N_PC_), 19-9-6 in respectively I-II-III. Signal to residual ratios (SRR) improvements for all reconstructed images are shown on the bottom. Optimal performance is obtained when using N_PC_=6.


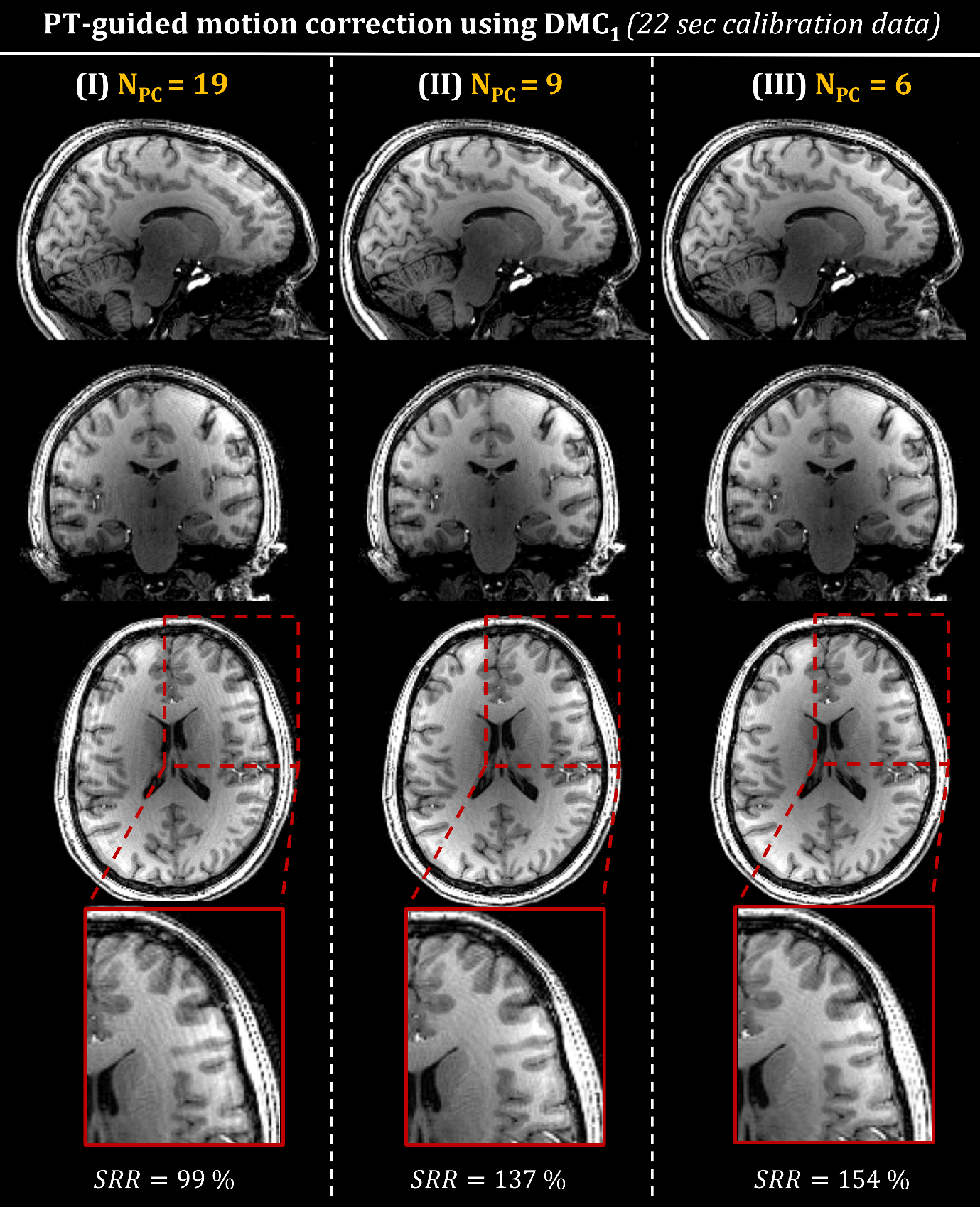


**Figure S4:** Boxplots (across HV) show the relative metric improvements of the PT-guided motion-corrected reconstructions using different levels of DMC data (A-B-C). Metrics are shown for the standard (I) MPRAGE and (II) SPACE sequences and are compared between the PT-*enforced* (hatched fill) and PT-*guided* motion correction. P-values of the statistical analysis between both reconstruction methods are shown between pairs of boxplots (paired sample tests were used).


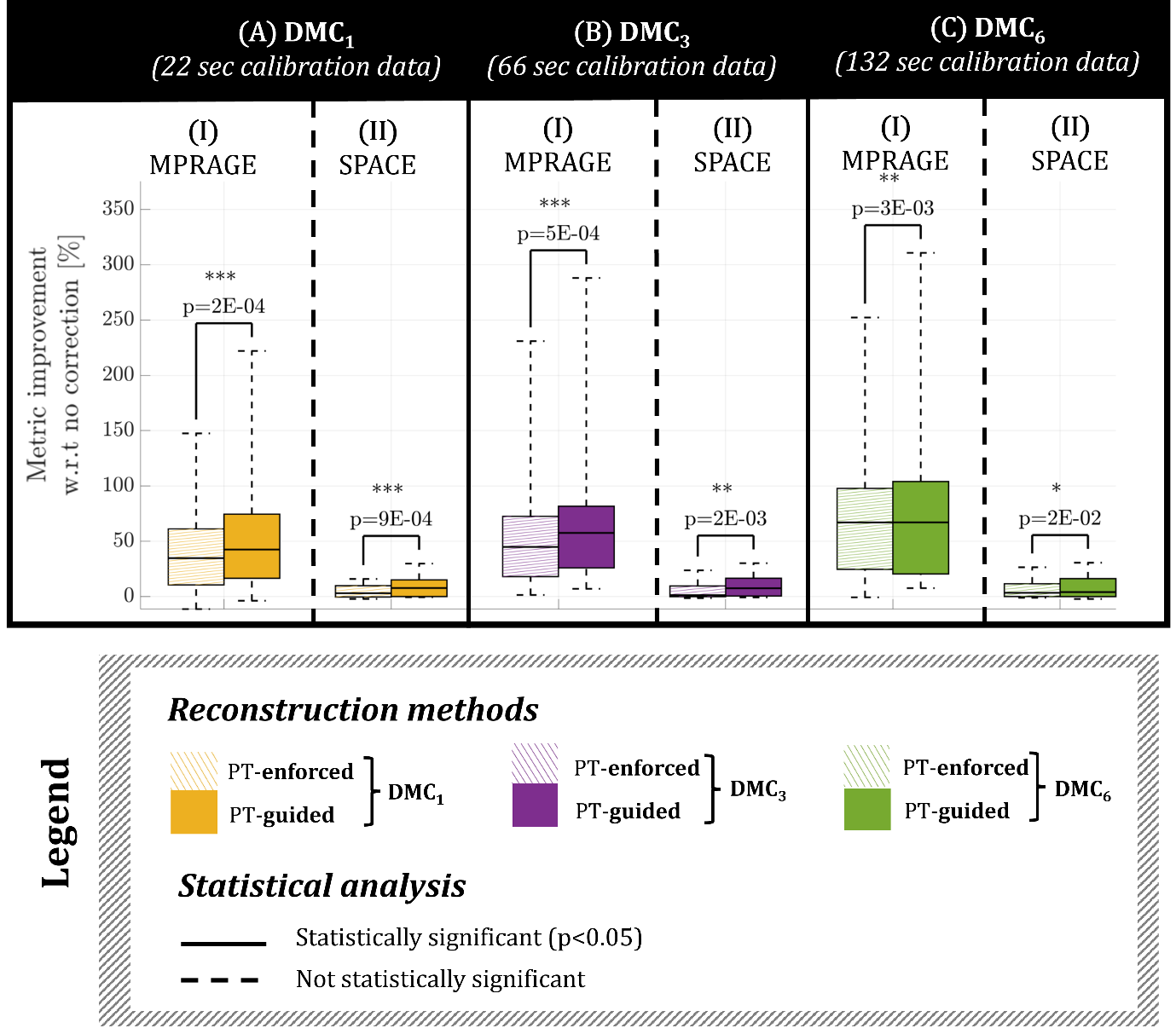


**Figure S5:** Boxplots (across HV) show the metric improvement of the different motion-corrected reconstructions. Results are presented for both motion experiments (I-II) individually and the P-values of the statistical analysis across DMC_1_/DMC_2_/DMC_3_ are shown (paired sample tests were used).


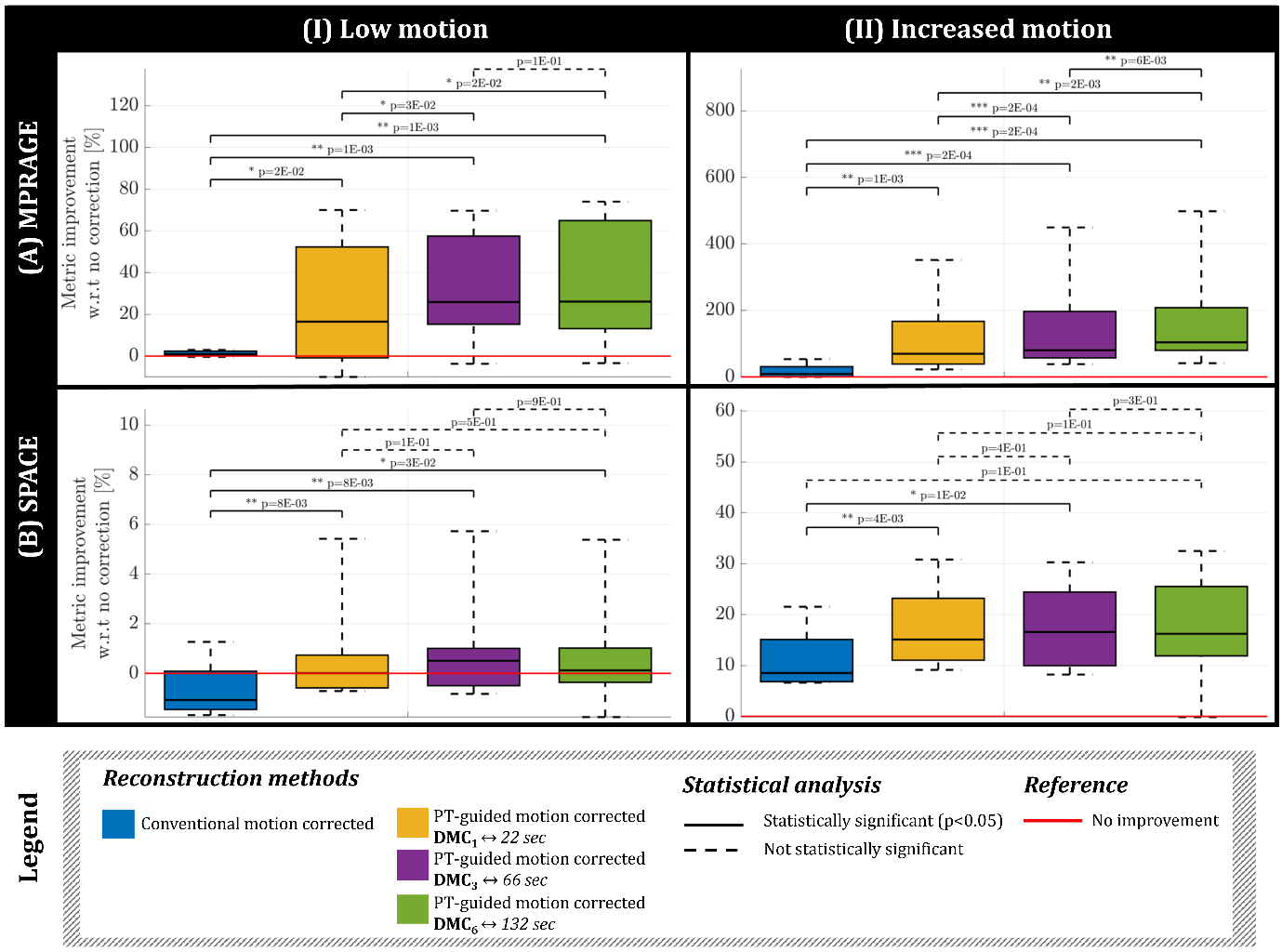


**Figure S6:** Image quality metrics **for each healthy volunteer (HV) individually** for the standard (I) MPRAGE and (II) SPACE sequences. Results from both the (A) low-motion and (B) increased motion experiment are shown. The signal to residual ratio (SRR) and normalized gradient squared (NGS) are shown using bar plots for MPRAGE and SPACE sequences. Bar plots contain the different reconstruction methods: uncorrected (red), conventional motion correction (blue) and the PT-informed motion correction (yellow-purple and green for calibration data corresponding to DMC_1_, DMC_3_ and DMC_6_ respectively). Both PT-enforced (hatched fill) and PT-guided (solid fill) motion corrections are shown.


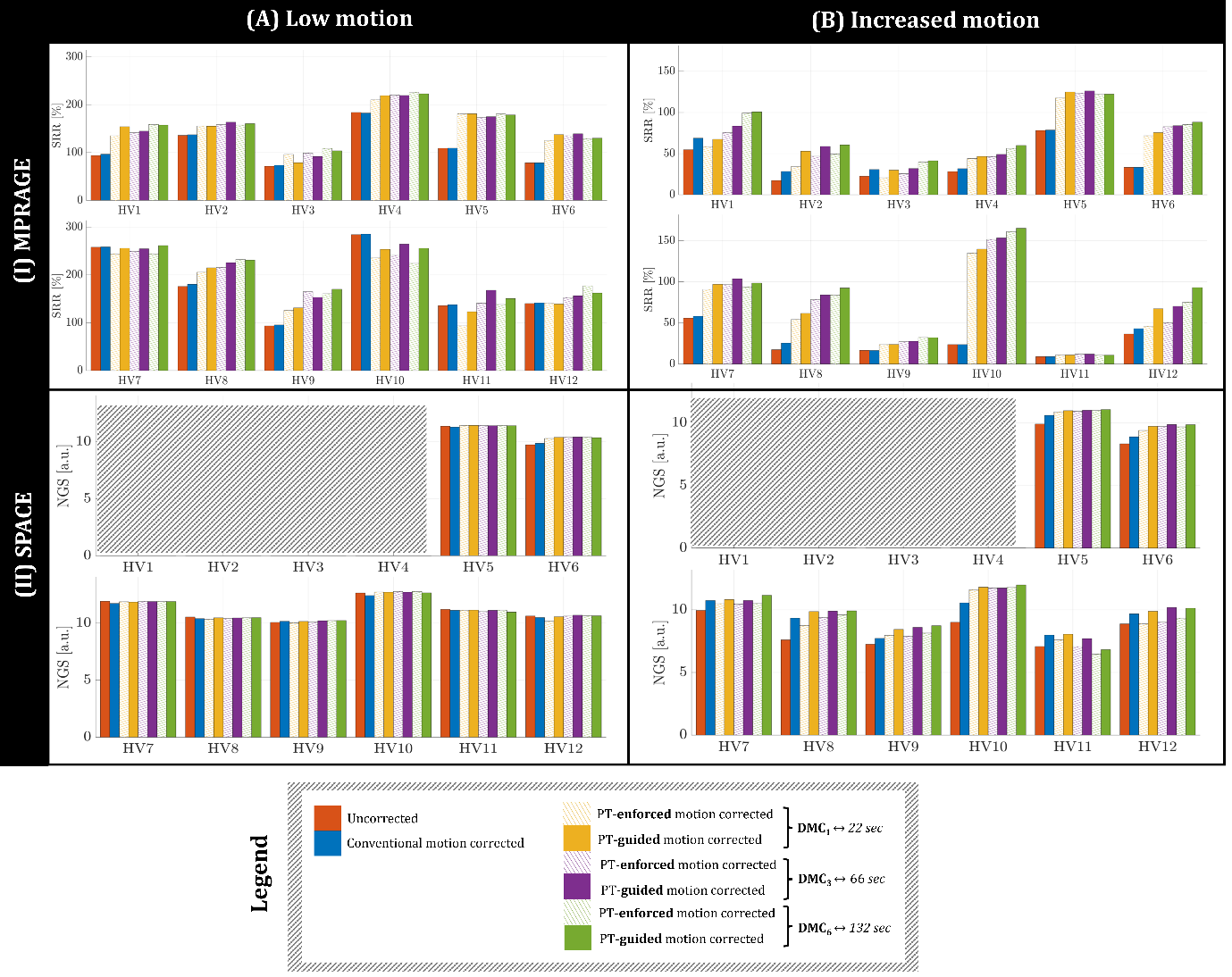


**Figure S7:** In-vivo PT signal for 2 healthy volunteers (rows) for the (A) MPRAGE and (B) SPACE sequences. PT signal is shown for acquisitions with (I) low and (II) increased instructed motion levels. Signal variations that are unlikely to arise from rigid head motion are indicated using red arrows (e.g. jumps, rapid oscillations).


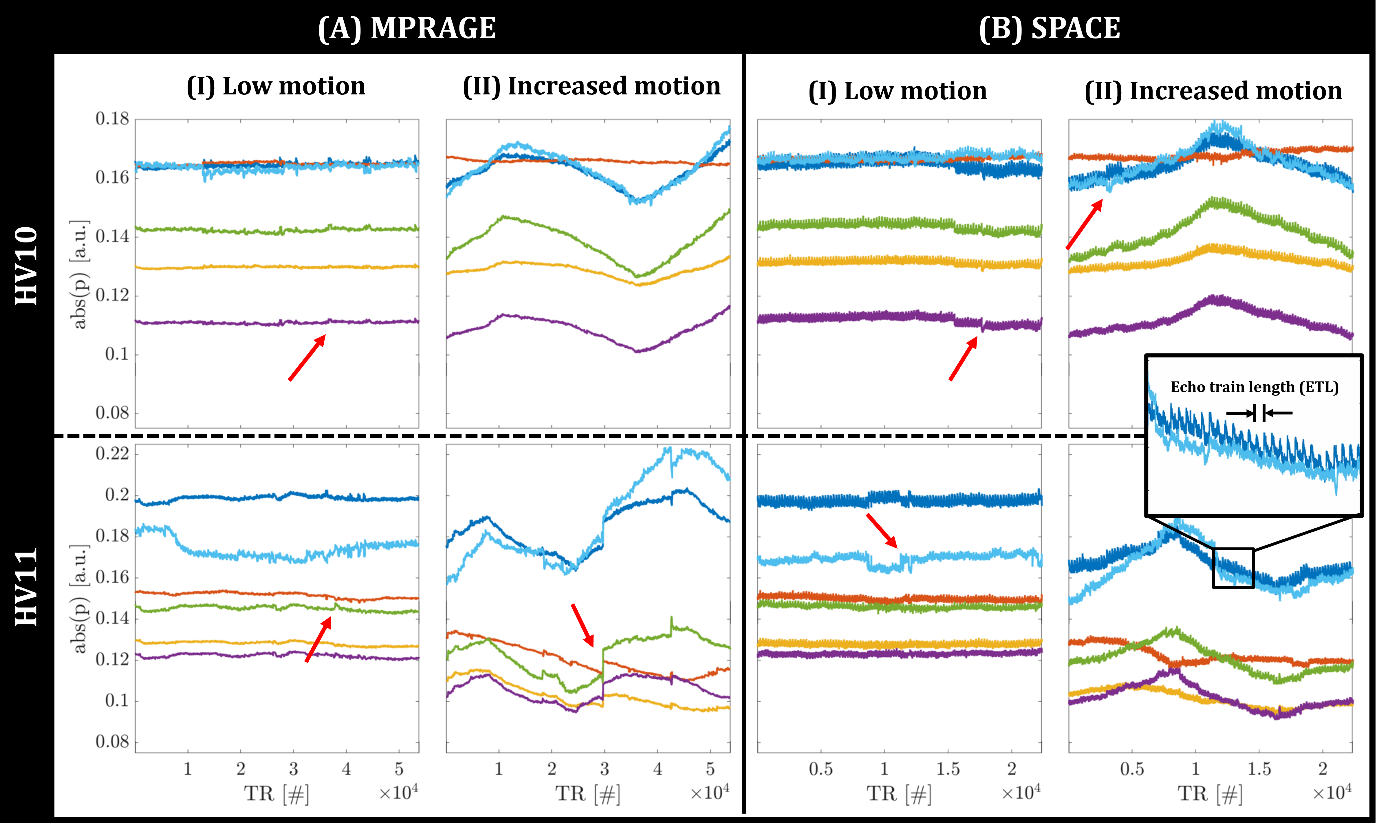

Supplement: Supporting Information [file EMS201791-supplement-Supporting_Information.docx]
