# Supplementary material for: Sequence-agnostic motion-correction leveraging efficiently calibrated Pilot Tone signals
Source: Magn Reson Med. Author manuscript; Available in PMC 2025 Feb 24. (PMC7617263; doi:10.1002/mrm.30161)
Supplement: Appendix [file EMS201791-supplement-Appendix.pdf]

SUPPORTING INFORMATION

Additional supporting information may be found in the online version of the article at the publisher's website.

Figure S1. Example of the "jumps" at the beginning and end of the acquired k-space readouts. The first 100 readouts (TRs) are shown for Coil 3 (same subject shown in Figure 3). Magnitude (*top*) and phase (*bottom*) of the measured k-space ($\boldsymbol{y}_{\mathrm{TR}_{\mathrm{meas}}}$) are shown. The jumps in the magnitude are indicated (*red*) and have a width of about three readout samples. The potential effect of these jumps on the phase is not visible due to the strong Pilot Tone (PT)–induced phase rolls throughout the readout.

Figure S2. Experiment 2: Validation errors in translation (A) and rotation (B) are shown for a range of distributed motion calibration (DMC) extractions (I–VI). For increased amount of calibration data (*lower rows*), the optimal N_{PC} (*blue*) increases, with lower errors for both translation and rotation. Additionally, the strong dependency on N_{PC} reduces when increasing the total amount of calibration data.

Figure S3. Pilot Tone (PT)–guided motion corrections for the standard MPRAGE in Healthy Volunteer 2 using distributed motion calibration (DMC_1). Reconstructions are shown for a range of principal components (N_{PC}): 19-9-6 in I-II-III, respectively. Signal to residual ratio (SRR) improvements for all reconstructed images are shown on the bottom. Optimal performance is obtained when using $N_{\mathrm{PC}} = 6$.

Figure S4. (A–C) Boxplots (across healthy volunteers [HVs]) show the relative metric improvements of the Pilot Tone (PT)–guided motion-corrected reconstructions using different levels of distributed motion calibration (DMC) data. Metrics are shown for the standard MPRAGE (I) and SPACE (II) sequences and are compared between the PT-*enforced* (*hatched fill*) and PT-*guided* motion correction. The signal to residual ratio (SRR) and normalized gradient squared (NGS) metrics are used for the MPRAGE and SPACE acquisitions, respectively. *p*-Values of the statistical analysis between both reconstruction methods are shown between pairs of boxplots (paired sample tests were used).

Figure S5. Boxplots (across healthy volunteers [HVs]) show the metric improvement of the different motion-corrected reconstructions. The signal to residual ratio (SRR) and normalized gradient squared (NGS) metrics are used for the MPRAGE and SPACE acquisitions, respectively. Results are presented for both motion experiments individually (I–II), and the *p*-values of the statistical analysis across distributed motion calibration $\mathrm{DMC}_1/\mathrm{DMC}_2/\mathrm{DMC}_3$ are shown (paired sample tests were used).

Figure S6. Image quality metrics for each healthy volunteer (HV) individually for the standard MPRAGE (I) and SPACE (II) sequences. Results from both the low-motion (A) and increased motion (B) experiment are shown. The signal to residual ratio (SRR) and normalized gradient squared (NGS) are shown using bar plots for MPRAGE and SPACE sequences. Bar plots contain the different reconstruction methods: uncorrected (*red*), conventional motion correction (*blue*), and the PT-informed motion correction (*yellow, purple, and green for calibration data corresponding to* DMC_1, DMC_3 *and* DMC_6, *respectively*). Both Pilot Tone (PT)–enforced (*hatched fill*) and PT-guided (*solid fill*) motion corrections are shown.

Figure S7. In vivo Pilot Tone (PT) signal for 2 healthy volunteers (*rows*) for the MPRAGE (A) and SPACE (B) sequences. PT signal is shown for acquisitions with low (I) and increased (II) instructed motion levels. Signal variations that are unlikely to arise from rigid head motion are indicated using red arrows (e.g., jumps, rapid oscillations).

How to cite this article: Brackenier Y, Cordero-Grande L, McElroy S, et al. Sequence-agnostic motion-correction leveraging efficiently calibrated Pilot Tone signals. *Magn Reson Med*. 2024;92:1881-1897. doi: 10.1002/mrm.30161

APPENDIX A. LEVENBERG–MARQUARDT UPDATES FOR THE CALIBRATION PARAMETERS

The Levenberg–Marquardt (LM) optimization in Eq. (6b) consists of estimating the calibration matrix ($\boldsymbol{C}$)

15222594, 2024, 5, Downloaded from https://onlinelibrary.wiley.com/doi/10.1002/mrm.30161 by Shaihan Malik - Kings College London , Wiley Online Library on [09/12/2024]. See the Terms and Conditions (https://onlinelibrary.wiley.com/terms-and-conditions) on Wiley Online Library for rules of use; OA articles are governed by the applicable Creative Commons License

from k-space acquisitions as follows:

$$\begin{aligned} \boldsymbol{C}^{i+1} &= \text{argmin}_{\boldsymbol{C}} \sum_{n\prime=1:N} \left|\left| \boldsymbol{A}_{n\prime} \mathbf{FST}\left(\boldsymbol{C}\boldsymbol{p}_{n\prime}\right)\boldsymbol{x} - \boldsymbol{y}_{n\prime} \right|\right|_2^2 \\ &= \text{argmin}_{\boldsymbol{C}} L(\boldsymbol{C}) \end{aligned} \tag{A1}$$

where L is the data-consistency loss function.

For ease of use, the calibration model is rewritten to represent $\boldsymbol{C}$ as a column ($\boldsymbol{C} \in \mathbb{R}^{M\times1}$), as follows:

$$\boldsymbol{z}_n = \boldsymbol{C}\boldsymbol{p}_n \rightarrow \boldsymbol{z}_n = \boldsymbol{p}'_n \boldsymbol{C}' \tag{A2}$$

where $M = 6 \times N_C$ and $\boldsymbol{p}\prime_n$ now represent a sparse matrix containing the Pilot Tone (PT) signals ($\boldsymbol{p}'_n \in \mathbb{R}^{6\times M}$). Using Eq. (A2), Eq. (A1) is reformulated as follows:

$$\begin{aligned} \boldsymbol{C}'^{i+1} &= \text{argmin}_{\boldsymbol{C}'} \sum_{n\prime=1:N} \left|\left| \boldsymbol{A}_{n\prime} \mathbf{FST}\left(\boldsymbol{p}'_{n\prime}\boldsymbol{C}'\right)\boldsymbol{x} - \boldsymbol{y}_{n\prime} \right|\right|_2^2 \\ &= \text{argmin}_{\boldsymbol{C}'} L\left(\boldsymbol{C}'\right) \end{aligned} \tag{A3}$$

By defining the gradient of L with respect to the calibration parameters ($\boldsymbol{C}'$) as $\boldsymbol{\nabla}^L_{\boldsymbol{C}'}$, the calibration update is given by

$$\boldsymbol{C}'^{i+1} = \boldsymbol{C}' - \left(\lambda^i_{\boldsymbol{C}'} + \boldsymbol{\nabla}^{L\ H}_{\boldsymbol{C}'} \boldsymbol{\nabla}^L_{\boldsymbol{C}'}\right)^{-1} \boldsymbol{\nabla}^L_{\boldsymbol{C}'} \tag{A4}$$

where $\lambda^i_{c'}$ is the LM hyperparameter that is updated every iteration.[29] Note that Eq. (A4) does not include a Jacobian, as the Jacobian is identical to the gradient $\boldsymbol{\nabla}$ for scalar functions like L ($L\left(\boldsymbol{C}'\right) : \mathbb{R}^{M\times1} \rightarrow \mathbb{R}$).

Elements of $\boldsymbol{\nabla}^L_{\boldsymbol{C}'}$ can be rewritten using the following chain rule:

$$\nabla^L_{C'_l} = \frac{\partial L}{\partial C'_l} = \sum_{n\prime} \sum_{q=1:6} \frac{\partial L}{\partial z_{n\prime,q}} \frac{\partial z_{n\prime,q}}{\partial C'_l} \tag{A5}$$

where the implementation of $\frac{\partial L}{\partial z_{n\prime,q}}$ can be found in Cordero-Grande et al.[29] and where

$$\frac{\partial z_q}{\partial C'_l} = \frac{\partial \sum_{\boldsymbol{v}} p'_{q,v} C'_{\boldsymbol{v}}}{\partial C'_l} = p'_{q,l} \tag{A6}$$

Once the update $\boldsymbol{C}'^{i+1}$ in Eq. (A4) is computed, it can be converted to its original form $\boldsymbol{C}^{i+1}$ used in Eq. (A1).

15222594, 2024, 5, Downloaded from https://onlinelibrary.wiley.com/doi/10.1002/mrm.30161 by Shaihan Malik - Kings College London , Wiley Online Library on [09/12/2024]. See the Terms and Conditions (https://onlinelibrary.wiley.com/terms-and-conditions) on Wiley Online Library for rules of use; OA articles are governed by the applicable Creative Commons License
